# *In-Vitro* Analysis of the Effect of Constructional Parameters and Dye Class on the UV Protection Property of Cotton Knitted Fabrics

**DOI:** 10.1371/journal.pone.0133416

**Published:** 2015-07-29

**Authors:** Chi-wai Kan, Chui-ha Au

**Affiliations:** Institute of Textiles and Clothing, The Hong Kong Polytechnic University, Hung Hom, Kowloon, Hong Kong; Federal University of Rio de Janeiro, BRAZIL

## Abstract

Cotton knitted fabrics were manufactured with different yarn types (conventional ring spun yarn and torque-free ring spun yarn) with different fibre types (combed cotton and combed Supima cotton) and yarn fineness (Ne30 and Ne40). These fabrics were then dyed with three types of dye (reactive, direct and sulphur dye) with three dye concentrations (0.1%, 1.0% and 5.0% on-weight of fabric (owf)) in three colours (red, yellow and blue). This study examined the impact of constructional parameters and dyeing on ultraviolet (UV) protection properties of cotton knitted fabric. *In-vitro* test with spectrophotometer was used for evaluating the UV protection property of dyed cotton knitted fabrics. Among the six parameters investigated, fineness of yarn and dye concentration were the most significant factors affecting UPF while the color effect is the least significant. Experimental results revealed that the UPF value of dyed fabrics made from combed cotton is generally higher than the combed Supima cotton since combed cotton is composed of shorter fibres which facilitate the blocking or absorption of UV radiation. Second, fabrics made with twist yarn (i.e. ring spun yarn) have higher UPF value than the corresponding ESTex one (i.e. torque-free yarn) in general since fabrics made with ring spun yarn tend to shrink during wet processing and so it is more compact. Third, the UPF value of fabrics made with 30Ne yarn was higher than the 40Ne one since it is thicker and has lower fabric porosity. Fourth, fabrics dyed with lower concentration of dye gave the lowest UPF. Fifth, the sulphur dyed samples performed worse than the reactive and direct dyed samples in terms of UV protection property. Sixth, there is no significant difference in UPF for red, yellow and blue coloured fabrics. Seventh, this study also demonstrated that lightness of fabric is negatively related to UV protection property.

## Introduction

Extant research has showed that ultraviolet radiation (UVR) from the Sun can be a primary cause of skin cancer [1, 2], including both non-melanoma and melanoma skin cancers [3]. Clothing has long been considered as a valuable means of protection against ultraviolet (UV) radiation [4]. Many researchers have studied various fabric parameters that influence UVR transmission including fibre composition [5–8], fabric construction [8–13], yarn twist [14], thickness [5, 7, 15], weight [15], wetness or moisture content [16, 17], stretch or extensibility [16, 18], chemical treatment or additives and coloration [19–23]. However, most of the studies have concentrated on woven fabrics only; few studies have examined knitted fabrics. In summer time, there is a higher chance of UVR exposure in terms of intensity and duration while cotton knitted garments are much more popular in that season. In previous research, the effect of cotton knitted fabric parameters and structures on UV protection has been studied [24, 25]. However, no systematic research on the effect of different dye classes on UV protection offered by cotton knitted fabric has been reported. Therefore, the aim of this study is to examine the ability of cotton knitted fabric dyed with different dye classes such as reactive, direct and sulphur dye to provide protection against UV radiation. In addition, the effect of fibre type, yarn count and spinning methods is also being studied. The ultraviolet protection factor (UPF) is used as a measuring parameter of the UV protection. In order to evaluate the UV protection property of the dyed cotton knitted fabrics, *In vitro* method is widely adopted for evaluating UV protection of textile using spectrophotometer. The UV protection ability of fabrics is commonly expressed in terms of ultraviolet protection factor (UPF). The UPF is calculated from the ratio of the UVR transmitted through air to the UVR transmitted though the fabric over a wavelength ranges from 290–400 nm. The calculated UPF values are usually rounded into a multiple of five and values higher than 50 are generally indicated as 50+. The high the UPF value, the better will be the UV protection property.

## Experimental

### Knitted fabrics and fabric preparation

Eight different types of cotton fabrics were used, knitted with four different types of yarns (combed cotton, combed Supima cotton, combed cotton ESTex and combed Supima cotton ESTex, sponsored by Central Textiles Limited, Hong Kong) in two different yarn counts (Ne30, Ne40), as shown in Table 1. These fabrics were knitted by Stoll CMS 822 E7.2 computerized flat knitting machine of gauge 14. Detailed information about the thickness and porosity of these fabrics is shown in Table A and Table B in S1 File, respectively.

**Table 1:** Specifications of the Fabric Samples.

Type of fabric	Yarn type	Yarn count	Weight (g/m^2^)	Thickness (mm)	Courses per inch (CPI)	Wales per inch (WPI)
Plain Knit	combed cotton^	Ne30	197.8	1.09	31	23
	combed cotton^	Ne40	158.6	1.03	26	24
	combed cotton ESTex*	Ne30	166.6	1.01	28	20
	combed cotton ESTex*	Ne40	134.7	0.92	24	22
	combed Supima cotton^	Ne30	196.3	1.02	28	20
	combed Supima cotton^	Ne40	131.5	0.88	24	22
	combed Supima cotton ESTex*	Ne30	162	0.91	28	21
	combed Supima cotton ESTex*	Ne40	121	0.81	23	21

^ Yarn refers to conventional ring spun yarn which is lableled as “Twist” in data analysis.

* Yarns labelled with “ESTex” is a type of torque-free ring spun yarn.

Fabrics were scoured and bleached by hydrogen peroxide (50%) (12ml/L), detergent (Sandopan DTC) (0.5g/L), sodium silicate (0.5g/L) and stabilizer AWN (0.5g/L) and sodium hydroxide (10g/L) was added to the liquor until the pH reached 10. The fabrics were scoured and bleached in the same bath for 60 minutes. The liquor-to-goods ratio was 50:1. After scouring and bleaching, the fabrics were rinsed thoroughly first with hot water and then cold water. Finally, the fabrics were neutralized with cold diluted sulphuric acid solution (0.5%). The fabrics were rinsed again with tap water, until they were free from acid and were air dried. The fabrics were conditioned under standard condition (relative humidity: 65±2%; temperature: 20±2°C) for at least 24 hours before use.

### Dyeing process

#### Dyes

Fabrics were dyed with three dye classes (reactive dye, direct dye and sulphur dye) (Clariant, Hong Kong). The dyes were used as received, without further purification. The fabrics were dyed with primary colours, red, yellow and blue with dye concentrations of 0.1%, 1% and 5% (concentration of dye used is in terms of on-weight of fabric (owf)). The dye specifications are listed in Table 2. The dyeing process was carried out in an oscillating dyeing machine (Tung Shing Dyeing Machines Factory Ltd, Hong Kong, China).

**Table 2:** Dye Specifications.

Sample code	Dye classes	Dye name
R-R	Reactive dye	Drimaren Red K-4BL
R-Y	Reactive dye	Drimaren Yellow K-2R
R-B	Reactive dye	Drimaren Blue K-2RL
D-R	Direct dye	Indosol Rubinole SF-RGN
D-Y	Direct dye	Indosol Yellow SF-2RL
D-B	Direct dye	Indosol Blue SF-2G 400
S-R	Sulphur dye	Diresul Red RDT-BG
S-Y	Sulphur dye	Diresul Yellow RDT-E
S-B	Sulphur dye	Diresul Blue RDT-2G 150

#### Reactive dye dyeing

The liquor-to-goods ratio of each dyebath was 100:1. Auxiliaries used for dyeing different reactive dye concentrations are shown in Table 3. The dyebath was set-up at 30°C with the fabric and sodium sulphate and the dyeing was run for 10 minutes. Then the dyebath temperature was increased constantly from 30°C to 60°C within 20 minutes. When the dyebath temperature reached 60°C, reactive dye was added and the temperature was maintained at 60°C for further 55 minutes. Then, sodium carbonate was added and the dyebath temperature of 60°C was maintained for a further 75 minutes. Dyed samples were taken out and rinsed with running water. Finally, soaping with detergent was conducted for 15 minutes at 90°C. Samples were dried in air. All samples were conditioned under standard condition (relative humidity: 65±2%; temperature: 20±2°C) for at least 24 hours prior to further evaluation.

**Table 3:** Auxiliaries used for Dyeing.

**Reactive dye**				
Dye concentration	Sodium sulphate (g/L)			Sodium carbonate (g/L)
0.10%	30			3
1%	45			4
5%	80			7
**Direct dye**				
Dye concentration	Sodium sulphate (g/L)			
	First addition			Second addition
0.10%	2.5			2.5
1%	7.5			7.5
5%	15			15
**Sulphur dye**				
Dye concentration	Sodium sulphate (g/L)			Sodium hydroxide (g/L)
0.10%	20	10	10	1
1%	20	10	10	1
5%	20	10	10	1

#### Direct dye dyeing

The liquor-to-goods ratio of the dyebath was 100:1. Auxiliaries used for different concentrations were as in Table 3. The dyebath was set-up at 40°C with fabric and sodium sulphate and was run for 10 minutes. The direct dye was added to the dyebath and temperature was maintained at 40°C for a further 20 minutes. Then, temperature was increased to 95°C within 30 minutes. The dyeing was run for another 30 minutes at 95°C and then more sodium sulphate was added. After that the dyeing was run for a further 60 minutes and was then washed off with running water. Finally, soaping with detergent was conducted for 15 minutes at 90°C. Samples were dried by air. All samples were subjected to a standard conditioning environment (relative humidity: 65±2%; temperature: 20±2°C) for at least 24 hours prior to further evaluation.

#### Sulphur dye dyeing

The liquor-to-goods ratio of the dyebath was 100:1. Auxiliaries used for different dye concentrations were as shown in Table 3. The dyebath was prepared at 60°C with sodium hydroxide. After 10 minutes, fabric was added into the dyebath. Sulphur dye was then added to dyebath 20 minutes later and dyebath temperature was maintained at 60°C for a further 10 minutes. Then, temperature was increased to 75°C within 10 minutes. When temperature reached 75°C, sodium sulphate was added at 5 minutes intervals three times and the temperature was maintained for a further 30 minutes. After dyeing, washing-off was conducted with running water. Finally, soaping with detergent was conducted for 15 minutes at 90°C. Samples were dried by air. All samples were subjected to a standard conditioning environment (relative humidity: 65±2%; temperature: 20±2°C) for at least 24 hours prior to further evaluation.

### *In-vitro* UPF measurement

The *in-vitro* measurement of UV protection properties of fabrics was evaluated by the Australian/New Zealand standard (AS/NZS 4399) with a Varian Cary 300 Conc UV-visible spectrometer. The UV protection properties in terms of UV protection factor (UPF) and UV radiation transmittance (UVA and UVB) were measured by the spectrophotometer. Fabrics (size: 22 x 34 mm) were cut out from the middle of each piece. These fabrics were then mounted, without tension, on the slide frames for measurement. The UV spectrophotometer recorded the transmittance between 290 nm and 400 nm at every 5 nm. For each fabric sample, four measurements were taken and the mean UPF was calculated according to Eq (1) [26]. Table 4 shows the classification system for good sun protection according to AS/NZS 4399.

$$\text{UPF}=\frac{\sum_{290}^{400}{\text{E}}_{\lambda }\cdot S_{\lambda }\cdot \Delta_{\lambda }}{\sum_{290}^{400}{\text{E}}_{\lambda }\cdot {\text{S}}_{\lambda }\cdot {\text{T}}_{\lambda }\cdot \Delta_{\lambda }}$$ (1)

where

*S*_λ_ is the solar spectral irradiance (in Wm^-2^Nm^-1^),

*E*_λ_ is the erythemal spectral effectiveness from CIE 1987,

T_λ_ is the spectral transmission through the textile,

Δ_λ_ is the bandwidth (in nm), and

λ is the wavelength (in nm).

**Table 4:** AS/NZS 4399 UPF Classification System.

UPF Range	UV Protection Category	Effective UV radiation Transmission (%)
15–24	Good Protection	6.7–4.2
25–39	Very Good Protection	4.1–2.6
40–50, 50+	Excellent Protection	< 2.5

### CIE L*a*b* measurement

Colour appearance in terms of CIE L*a*b* values were measured by Macbeth CE-7000A spectrophotometer. Different shades were identified and were later compared with results of UV test. Colours are represented by CIE L*, a* and b* coordinates where L* represents lightness (from 0 (black) to 100 (white)), a* represents red-green (positive a* = red, negative a* = green) and b* yellow-blue (positive b* = yellow, negative b* = blue).

## Results and Discussion

UV protective property of textile materials depends on many factors, the most frequently cited being fibre composition, fabric construction, fabric cover factor, dye and finish on fabrics [27]. The dye used to colour a textile can affect the UV protective ability of a fabric, depending on the position and intensity of the UV wavelength absorption bands of the dye and the concentration of the dye in the textile [28]. In this study, six variables were examined its effect on UV protective property, including (i) types of fibres (combed, combed supima), (ii) yarn spinning method (twist, ESTex), (iii) yarn count (30Ne, 40Ne), (iv) dye concentration (0.1%, 1%, 5%), (v) dye class (reactive, direct, sulphur) and (vi) colour (red, yellow, blue). In order to determine the effect of these variables, analysis of variance (ANOVA) test was carried out by SPSS 19.0. The significance level of the statistical analysis conducted in this study was set at 0.05.

Before performing the ANOVA test, three assumptions were examined, (i) no significant outliers, (ii) normal distribution of the dataset and (iii) homogeneity of variance. By plotting the UPF data with boxplots in SPSS, fabrics with extreme UPF values (those that extend more than 3 box-lengths from the edge of the box in a boxplot) were detected and eventually thirteen fabrics were eliminated for further investigation. After that, the normality of the data in each group was investigated and this can be checked against the skewness value. Skewness value less than plus or minus one implies normal distribution of the data. Table C in S1 File shows that most of the skewness value is larger than 1, implying skewed UPF value (not normally distributed). However, ANOVA is quite robust to violation of normality, so this dataset can further process for the ANOVA test. Third, Table D in S1 File shows that the assumption of homogeneity of variances has been violated since Levene’s test is significant(p<0.05).

**Fig 1:**
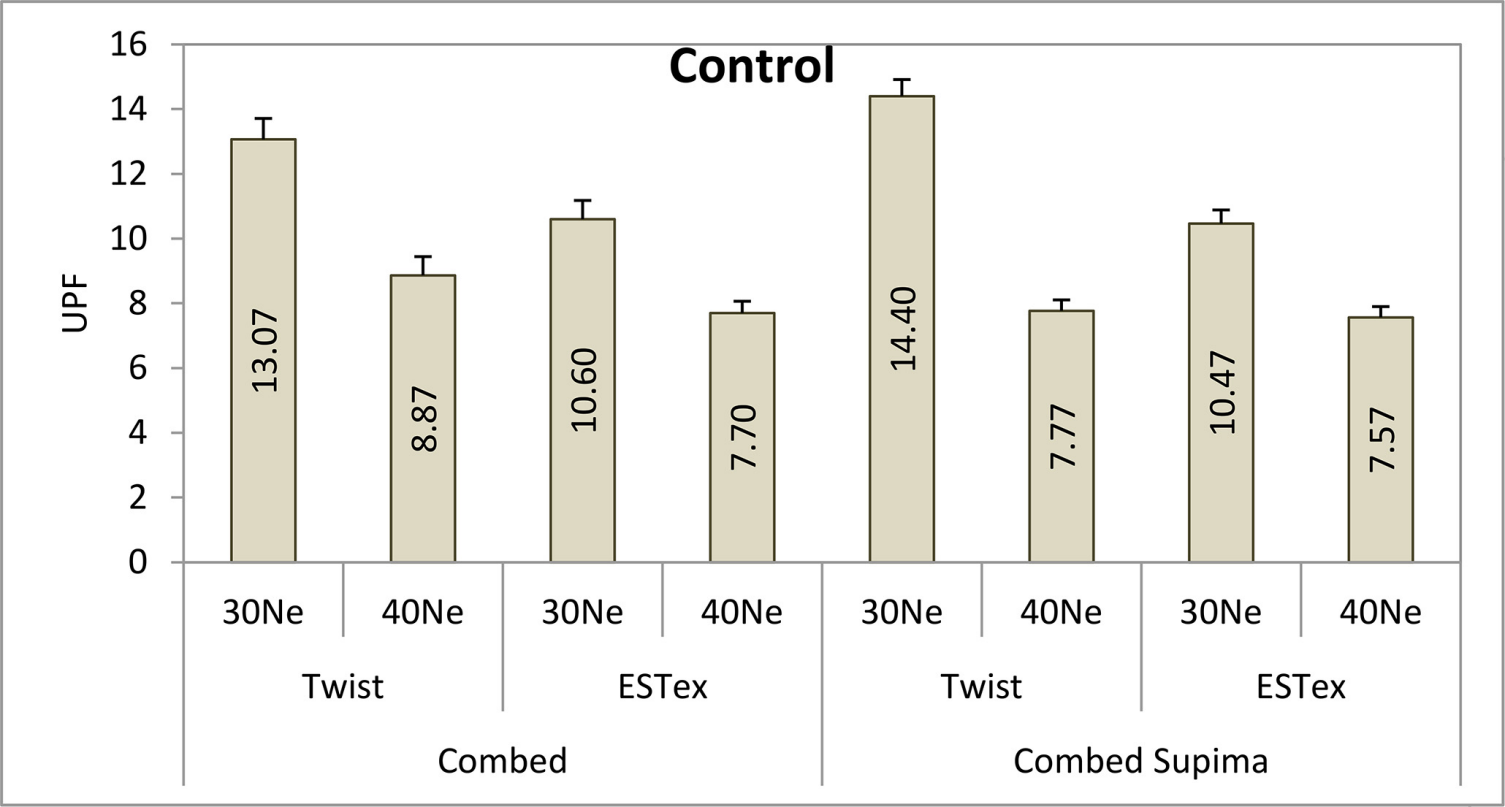
UPF values of various control fabrics (without dyeing).

**Fig 2:**
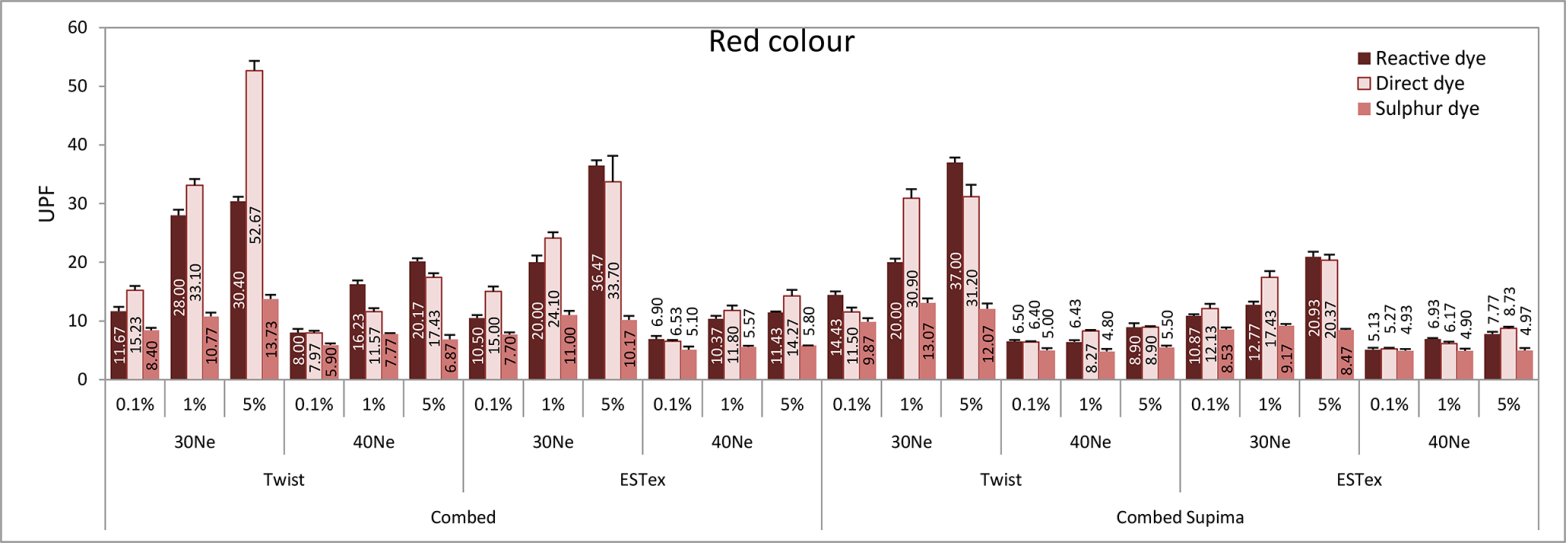
UPF values of various fabrics in red colour.

**Fig 3:**
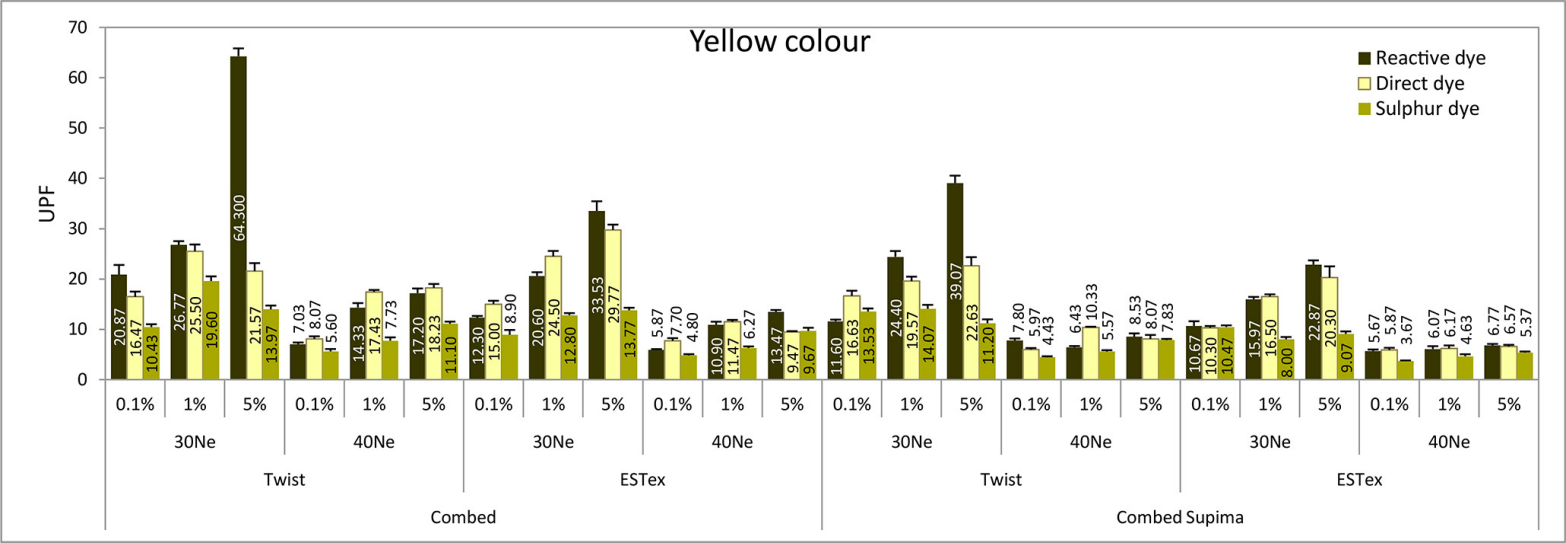
UPF values of various fabrics in yellow colour.

**Fig 4:**
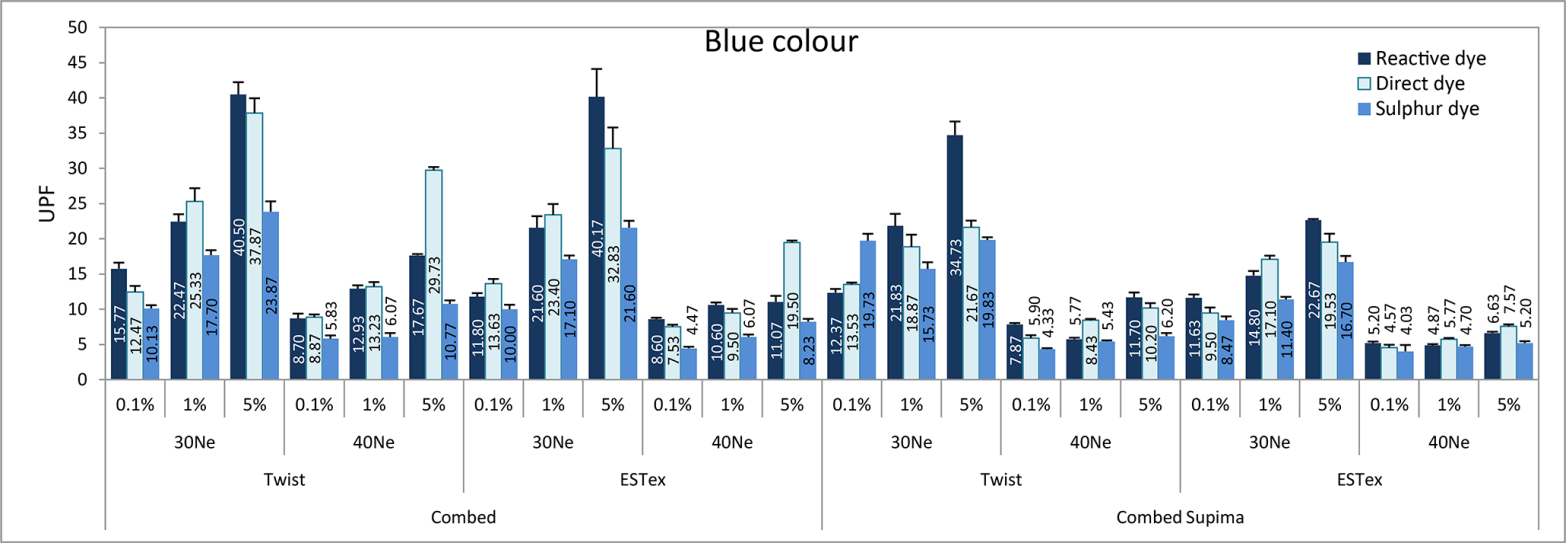
UPF values of various fabrics in blue colour.

The ANOVA results of the UPF property of fabrics are shown in Table E in S1 File. It shows that both main effect and interaction effect is significant. Eta is an indicator of the proportion of variance that is due to between groups differences. Among the six main effects, partial Eta squared for the effect of colour is the lowest. Only 23% of the variance in UPF can be predicted from colour. On the other hand, partial Eta squared for the effect of yarn finesness is the highest and around 98% of the variance in UPF can be predicted from yarn fineness. Accordingly, these fabrics were classified into four groups—control (before dyeing), red, yellow and blue fabrics and their UPF results are shown in Figs 1–4.

In general, it can be observed that the UPF for the fabrics dyed with 0.1% dye solution is even lower than the control sample (i.e. without dye). It is understandable as fabric might shrink during the dyeing process, thus reducing fabric thickness (shown in Table A in S1 File).

Table E in S1 File 5 shows that the F-ratio for the main effect as well as the 2-way interaction effect is the most significant factor affecting UPF. The partial Eta squared of the 2-way interaction effect is quite high and is as high as 0.815 for Dye concentration*Colour. The partial Eta squared and F-ratio for the 3-way, 4-way, 5-way or 6-way interaction effect is generally lower, implying the variation in UPF is less likely to be affected by these higher-order interactions. As a result, these higher-interaction terms (i.e. 3-, 4-, 5- and 6-factor interactions) were pooled into the error term and the pooled ANOVA results was shown in Table F in S1 File. In the following discussion, main effects and two-way interaction effect were the focus. For the 2-way interaction, post hoc tests were performed to investigate the presence of significant difference between pairs. Since Levene’s test suggests that the UPF data violate the assumption of homogeneity of variances, Tamhane’s T2 test, which does not assume equal variances, was selected.

The F-ratio in Table F in S1 File shows that the magnitude of the main effect is great and is higher than the 2-way interaction effect. Fineness of yarn is the most significant factor varying the UPF (F = 3062.2), followed by dye concentration (F = 656.6), dye class (F = 486.6), types of fibers (F = 409.5), yarn spinning method (F = 234.2) and color (F = 6.9).

### Effect of fibre type on UV protection

**Fig 5:**
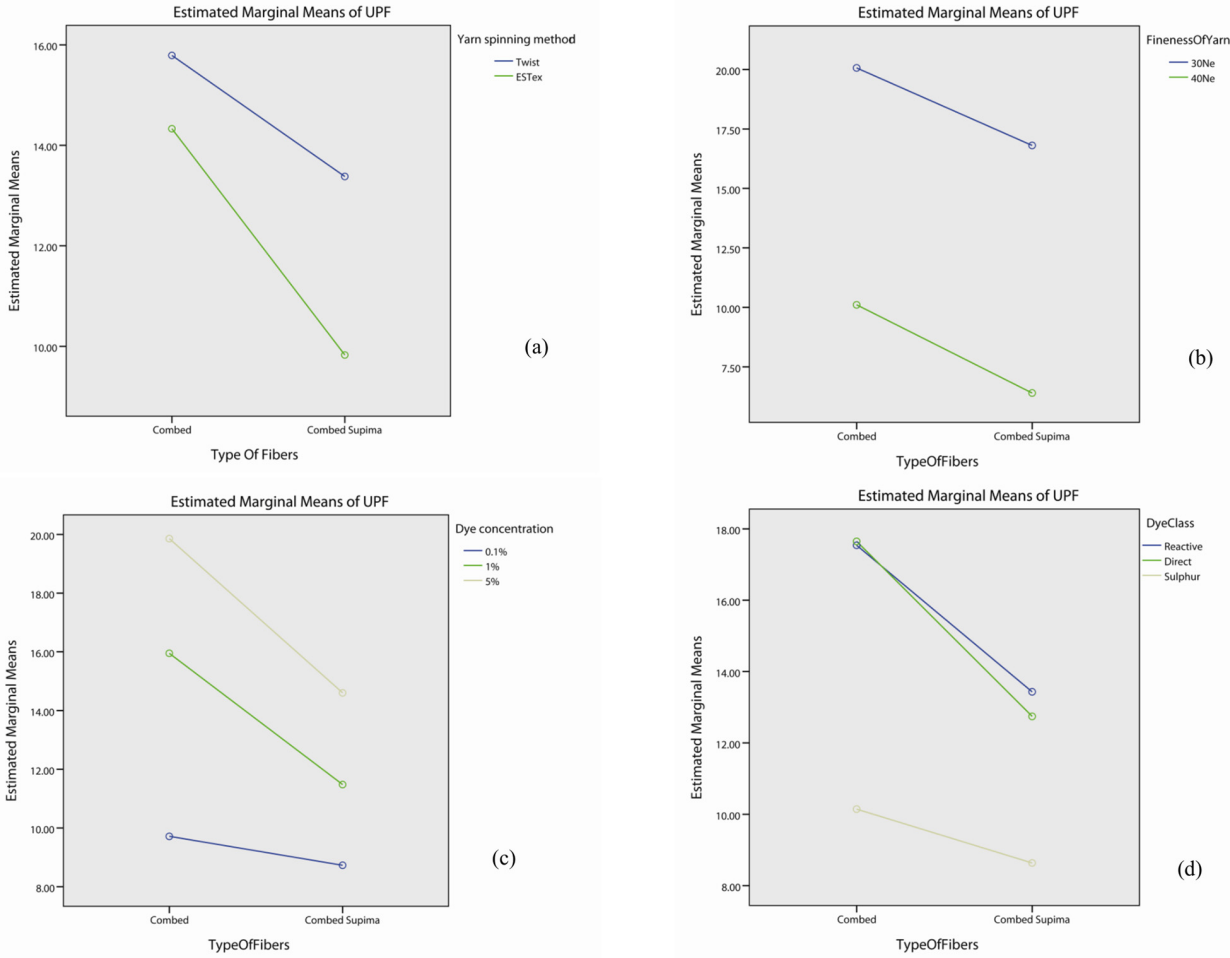
Profile plots showing the interaction effect of fibre type with (a) yarn spinning method, (b) fineness of yarn, (c) dye concentration, (d) dye class, and (e) colour.

The interaction effect of fibre type is significant with yarn spinning method (p<0.05), dye concentration (p<0.05), dye class (p<0.05) and colour (p<0.05), but not in yarn fineness (p>0.05) as shown in Table F in S1 File. The profile plots of fibre type with yarn spinning method (Fig 5(A)), fineness of yarn (Fig 5(B)), dye concentration (Fig 5(C)), dye class (Fig 5(D)) and colour (Fig 5(E)) show that the UPF value of fabrics made with combed fibre is generally higher than the one by combed Supima fibre. Although the UPF decreased from Combed to Comber Supima when fabrics were made either by conventional (lablled as “twist” during analysis) or ESTex yarn, 30Ne or 40Ne yarn or treated with different dye, different dye concentration or different colour, the degree of decrement is not the same. This explains the significant interaction observed. Table F in S1 File also shows that the effect of fibre type itself is significant (p<0.05).

Researchers have found that when comparing the UPF of the dry undyed fabrics made by Supima cotton fibre with the combed cotton fibre, combed Supima cotton fabrics provides better UPF rating than the combed cotton one when using the same spinning method [29]. In contrast with the previous study, the present work found that, the UPF of dyed knitted fabrics made from combed Supima cotton is generally worse than the combed cotton fabrics. Post hoc test, shown in Table 5, suggests that the combed fabric has better UPF property than the corresponding combed Supima fabrics irrespective of yarn spinning method (p<0.05) and yarn fineness (p<0.05). The superiority of combed fabric is also more prominent when the dye concentration is high. The UPF of fabrics made by combed fibre with 1% and 5% dye concentration is significantly higher than the corresponding fabrics made by combed Supima fibre (p<0.05). However, no significant difference was found for the fabrics in pale colour (0.1% dye concentration, p>0.05). In additional, combed fabrics dyed with reactive dye or direct dye has higher UPF property than the corresponding combed Supima fabrics (p<0.05). This finding, however, does not apply to the sulphur dyed samples (p>0.05). Moreover, the combed yellow and blue samples have higher UPF than the corresponding combed Supima fabrics (p<0.05), but it does not apply to the red samples (p>0.05).

**Table 5:** Results of Post hoc tests showing the comparisons of fabrics made with combed and combed Supima fibres under the interaction effect of other variables.

(I)	(J)	Mean Difference (I-J)	Std. Error	Sig.	95% Confidence Interval	
					Lower Bound	Upper Bound
Combed-twist	Combed Supima-twist	2.40931*	0.83233	0.024	0.209	4.6096
Combed-ESTex	Combed Supima-ESTex	4.38726*	0.69655	0	2.5444	6.2301
Combed-30Ne	Combed Supima-30Ne	9.86843*	0.68676	0	8.0492	11.6877
Combed-40Ne	Combed Supima-40Ne	3.70630*	0.31171	0	2.8803	4.5323
Combed-0.1%	Combed Supima-0.1%	0.98426	0.45756	0.389	-0.3667	2.3352
Combed-1%	Combed Supima-1%	4.47037*	0.81796	0	2.0552	6.8855
Combed-5%	Combed Supima-5%	5.10487*	1.20125	0	1.5559	8.6539
Combed-reactive dye	Combed Supima-reactive dye	3.95059*	1.09585	0.006	0.7145	7.1866
Combed-direct dye	Combed Supima-direct dye	4.90359*	0.94816	0	2.102	7.7052
Combed-sulphur dye	Combed Supima-sulphur dye	1.51019	0.55052	0.093	-0.1153	3.1357
Combed-Red	Combed Supima-Red	2.71316	0.99002	0.094	-0.2106	5.6369
Combed-Yellow	Combed Supima-Yellow	3.18746*	0.86713	0.004	0.627	5.7479
Combed-Blue	Combed Supima-Blue	4.23973*	0.99762	0	1.2913	7.1881

* The mean difference is significant at the 0.05 level.

The results from the present study (study on dyed samples) are opposite to previous finding (study on undyed samples). Combed yarn consists of short staple fibre while combed Supima yarn is composed of longer and finer staple fibre. During dyeing, dye might penetrate into the short staple fibres more easily than the longer staple fibres. As a result, knitted fabrics made from combed cotton fibre (short staple fibre) might readily block or absorb much UV radiation, thereby providing better UV protection.

### Effect of yarn spinning method on UV protection

**Fig 6:**
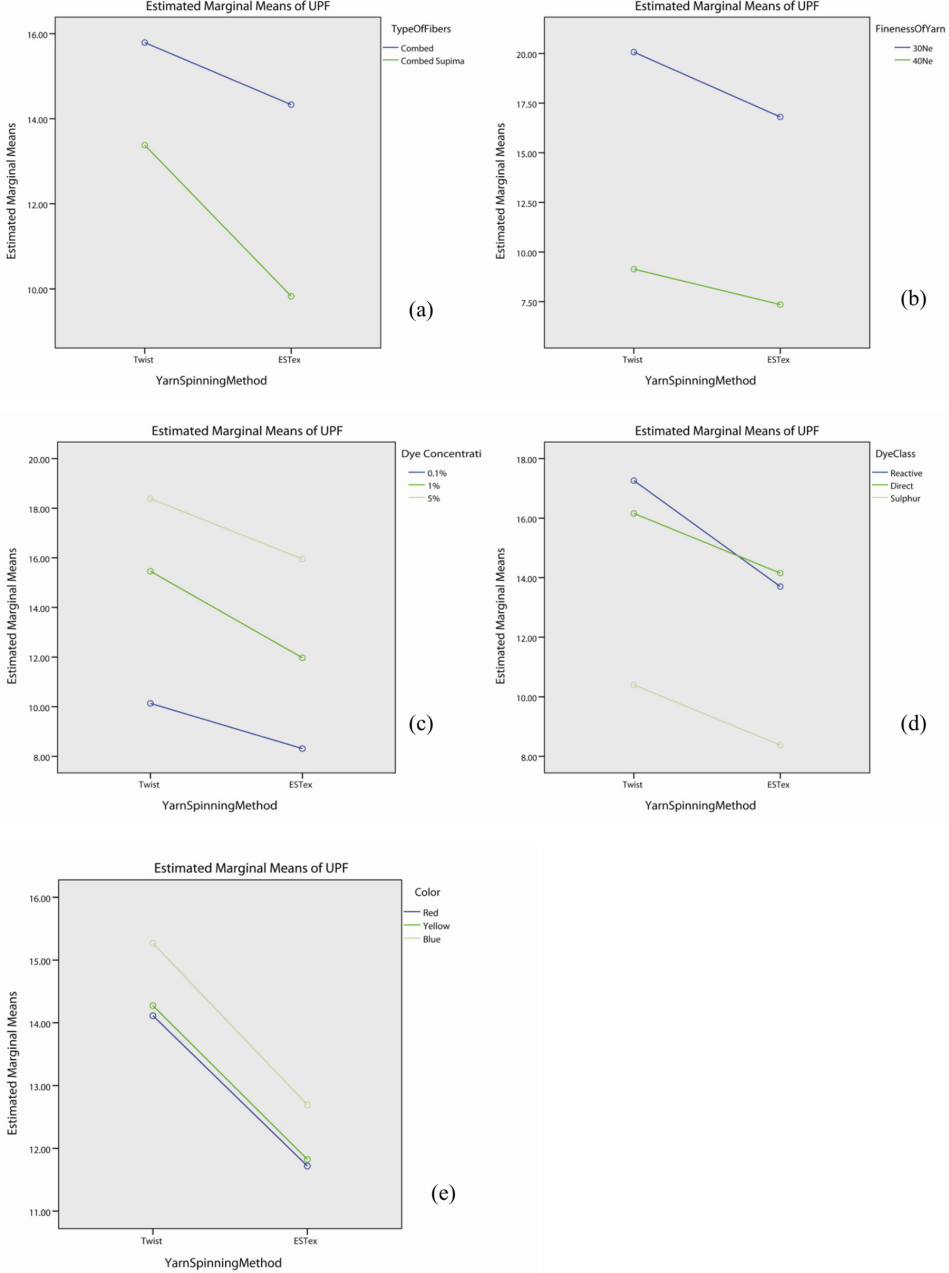
Profile plots showing the interaction effect of yarn spinning method with (a) types of fibre, (b) fineness of yarn, (c) dye concentration, (d) dye class, and (e) colour.

As shown in Table F in S1 File, the interaction effect of yarn spinning method is significant with fibre type (p<0.05), yarn fineness (p<0.05), dye concentration (p<0.05) and dye class (p<0.05), but not in colour (p>0.05). The profile plots of yarn spinning method with fibre type (Fig 6(A)), fineness of yarn (Fig 6(B)), dye concentration (Fig 6(C)), dye class (Fig 6(D)) and colour (Fig 6(E)) show that the UPF value of fabrics made with twist yarn is generally higher than the one by ESTex yarn. Although UPF decreases from Twist yarn to ESTex yarn when knitting different fibres, yarn or dyeing with different dye or dye concentration, the degree of decrement is different for each case. It explains the significant interaction observed in Table F in S1 File. Table F in S1 File also shows that the effect of yarn spinning method itself is significant (p<0.05).

Post hoc test, shown in Table 6, suggests that fabrics made with twist yarn have higher UPF value than the corresponding ESTex one irrespective of yarn fineness (p<0.05). ESTex yarn, a commercial torque-free ring spun yarn, is produced by a new spinning technology which can produce low twist yarns with balanced torque [30] but broadly the same strength [31–33]. Table 6 also shows that the UPF value of twist-combed Supima cotton fabric is significantly higher than the corresponding ESTex-combed Supima fabric (p<0.05). However, the UPF value of twist-combed cotton fabric does not have significant difference with the corresponding ESTex-combed fabric (p>0.05). As mentioned in the previous section, combed Supima fabric is composed of longer stable fibres. When the fibres were being twisted by ring spinning method, the twist will hold the fibres tighter in place. This effect is even more prominent in the combed Supima samples due to the length of the fibres. During wet treatments (dyeing), the residual torque in the yarn will be released. For those fabrics with twist held properly, the fabric might be distorted and shrunk. As a result, the knitted fabrics made from conventional ring spun yarn become more compact which helps resist penetration of UV rays [34]. This explains why twist-combed Supima fabrics got higher UPF than the ESTex-combed Supima fabrics.

**Table 6:** Results of Post hoc tests showing the comparisons of fabrics made with twist and ESTex yarn under the interaction effect of other variables.

(I)	(J)	Mean Difference (I-J)	Std. Error	Sig.	95% Confidence Interval	
					Lower Bound	Upper Bound
Twist-combed	ESTex-combed	1.5745	0.83132	0.305	-0.6232	3.7722
Twist-combed Supima	ESTex-combed Supima	3.55246*	0.69775	0	1.7065	5.3984
Twist-30Ne	ESTex-30Ne	3.36738*	0.79499	0	1.2657	5.4691
Twist-40Ne	ESTex-40Ne	1.78335*	0.3483	0	0.8623	2.7044
Twist-0.1%	ESTex-0.1%	1.82129*	0.4485	0.001	0.4961	3.1464
Twist-1%	ESTex-1%	3.48518*	0.83454	0.001	1.0198	5.9506
Twist-5%	ESTex-5%	2.58896	1.22917	0.424	-1.0423	6.2202
Twist-reactive dye	ESTex-reactive dye	3.73295*	1.10005	0.012	0.4836	6.9823
Twist-direct dye	ESTex-direct dye	2.00218	0.98205	0.478	-0.8983	4.9027
Twist-sulphur dye	ESTex-sulphur dye	2.02127*	0.54476	0.004	0.4122	3.6303
Twist-Red	ESTex-Red	2.39387	0.99401	0.223	-0.5423	5.33
Twist-yellow	ESTex-Yellow	2.44924	0.87661	0.08	-0.1394	5.0379
Twist-Blue	ESTex-Blue	2.75592	1.01252	0.099	-0.2347	5.7465

* The mean difference is significant at the 0.05 level.

Moreover, conventional ring spun combed cotton yarn is more hairy and coarser than the ESTex yarns. As a result, pores of the fabric are blocked by the hairiness and the short fibres. Consequently, the UV rays are scattered by the short fibres, resulting in increased UV protective properties. Although fabrics with conventional ring spun yarn got higher UPF value in this study, other fabric properties such as softness and smoothness may not be as good as ESTex fabric. Therefore, knitwear manufacturers should list out specific criteria and consider the comfort as well as protection properties when producing UV protective garments.

### Effect of yarn fineness on UV protection

**Fig 7:**
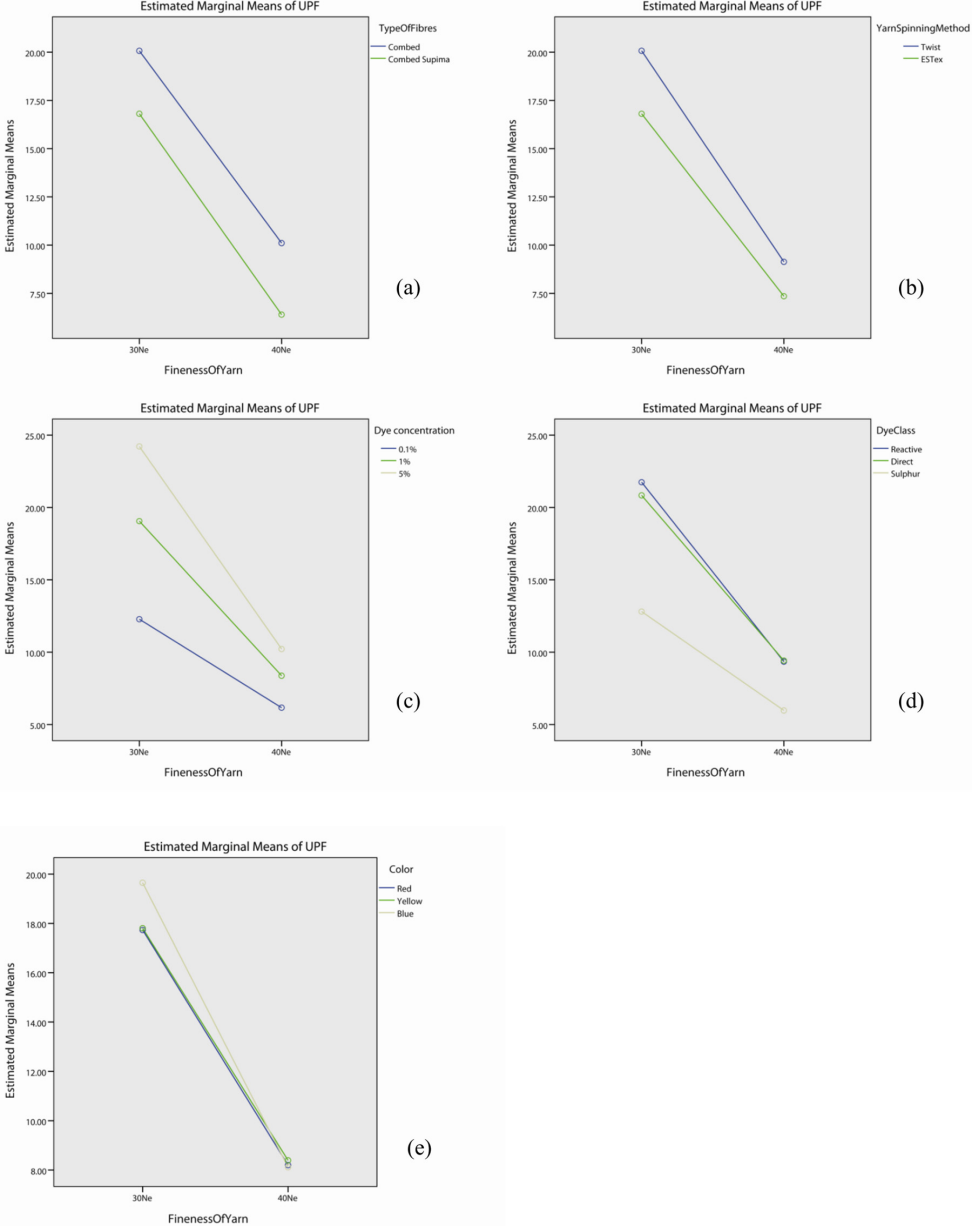
Profile plots showing the interaction effect of yarn fineness with (a) types of fibre, (b) yarn spinning method, (c) dye concentration, (d) dye class, and (e) colour.

As shown in Table F in S1 File, the interaction effect of yarn fineness is significant with yarn spinning method (p<0.05), dye concentration (p<0.05), dye class (p<0.05) and colour (p<0.05) but not in type of fibres (p>0.05). The profile plots of yarn fineness with fibre type (Fig 7(A)), yarn spinning method (Fig 7(B)), dye concentration (Fig 7(C)), dye class (Fig 7(D)) and colour (Fig 7(E)) show that the UPF value of fabrics made with 30Ne yarn is generally higher than the one by 40Ne yarn. Table F in S1 File also shows that the effect of yarn fineness is significant (p<0.05).

The post hoc tests, shown in Table 7, suggest that irrespective of fibre type, yarn spinning method, dye concentration, dye class and colour, the UPF value of the fabrics made with 30Ne yarn is significantly higher than the corresponding fabrics by 40Ne yarn. Fabrics made of yarn count Ne30 are thicker (as shown in Table A in S1 File) and have lower fabric porosity (as shown in Table B in S1 File) than fabrics made with Ne40 yarn. Fabrics with compact knitting structure allow less UV radiation transmittance through the fabric which results in higher UV protection.

**Table 7:** Results of Post hoc tests showing the comparisons of fabrics made with 30Ne and 40Ne yarn under the interaction effect of other variables.

(I)	(J)	Mean Difference (I-J)	Std. Error	Sig.	95% Confidence Interval	
					Lower Bound	Upper Bound
30Ne-combed	40Ne-combed	9.86843*	0.68676	0	8.0492	11.6877
30Ne-combed Supima	40Ne-combed Supima	10.40802*	0.51409	0	9.0441	11.7719
30Ne-Twist	40Ne-Twist	10.93340*	0.65337	0	9.2028	12.664
30Ne-ESTex	40Ne-ESTex	9.34937*	0.57134	0	7.8353	10.8634
30Ne-0.1%	40Ne-0.1%	6.11201*	0.28659	0	5.263	6.961
30Ne-1%	40Ne-1%	10.67779*	0.58333	0	8.9516	12.4039
30Ne-5%	40Ne-5%	13.87955*	0.9163	0	11.1616	16.5975
30Ne-reactive dye	40Ne-reactive dye	12.27166*	0.85903	0	9.7233	14.82
30Ne-direct dye	40Ne-direct dye	11.41236*	0.71165	0	9.3047	13.52
30Ne-sulphur dye	40Ne-sulphur dye	6.83056*	0.38459	0	5.6903	7.9708
30Ne-Red	40Ne-Red	9.53330*	0.83375	0	7.06	12.0066
30Ne-yellow	40Ne-Yellow	9.41403*	0.69432	0	7.3572	11.4709
30Ne-Blue	40Ne-Blue	11.39690*	0.76054	0	9.1425	13.6513

* The mean difference is significant at the 0.05 level.

### Effect of Dye concentration on UV protection

**Fig 8:**
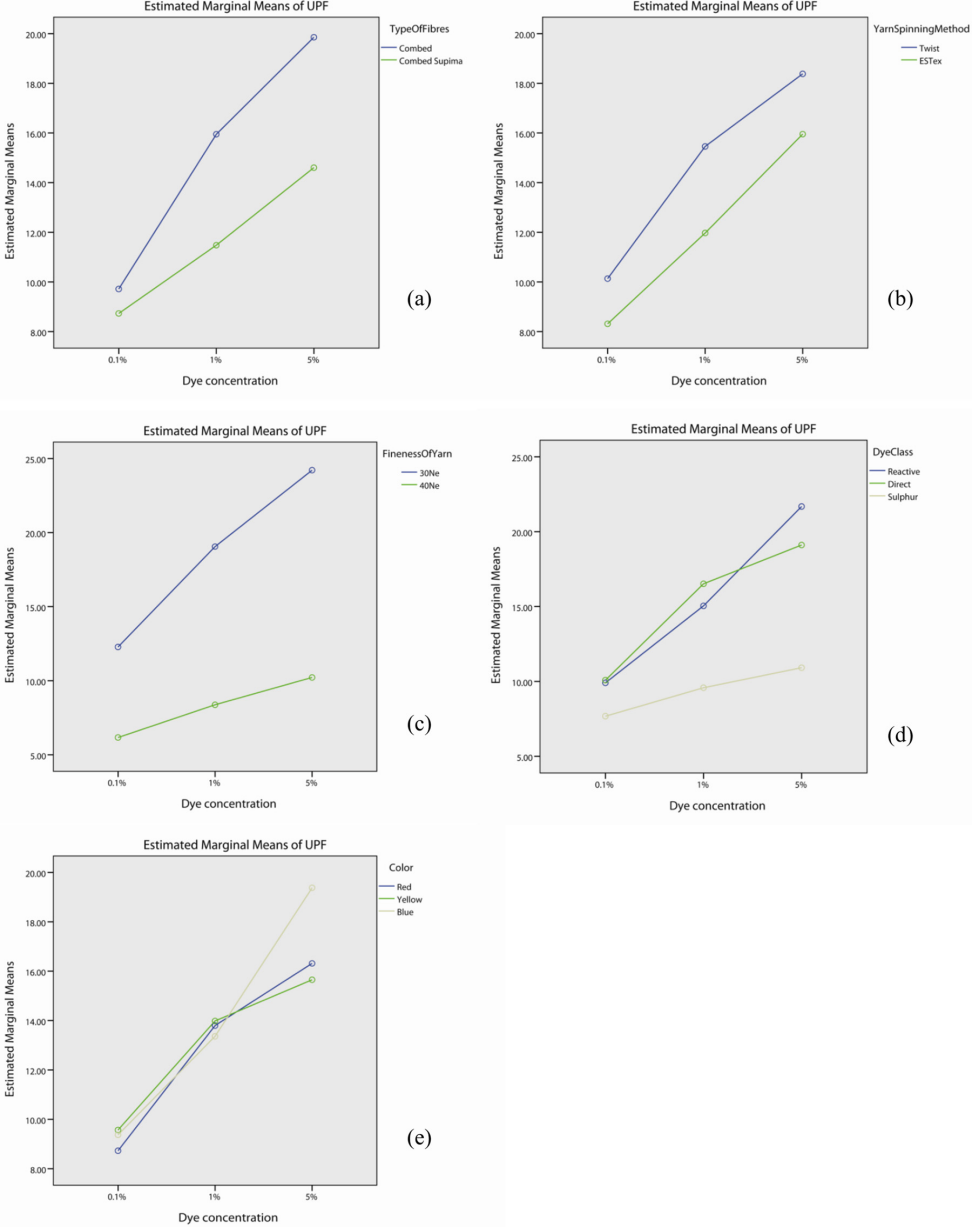
Profile plots showing the interaction effect of dye concentration with (a) types of fibre, (b) yarn spinning method, (c) yarn fineness, (d) dye class, and (e) colour.

Table F in S1 File demonstrates that the interaction effect of dye concentration is significant with fibre type (p<0.05), yarn spinning method (p<0.05), yarn fineness (p<0.05), dye class (p<0.05) and colour (p<0.05). The profile plots of dye concentration with fibre type (Fig 8(A)), yarn spinning method (Fig 8(B)), fineness of yarn (Fig 8(C)), dye class (Fig 8(D)) and colour (Fig 8(E)) show that the UPF value of fabrics dyed with 5% dye solution is generally higher than the one by 1% dye and 0.1% dye. Although the UPF increases with dye concentration, the slope has remarkable difference for different fibre type, yarn spinning methods, yarn fineness, dye class and color. Therefore, the 2-way interaction effect is significant. Table F in S1 File also shows that the effect of dye concentration itself is significant (p<0.05).

After dyeing, the UPF values increased gradually with increased dye concentration. Darker shades of the same hue were obtained with the increase in dye concentration. As shown in Table 8, the UPF results of combed fabrics treated with 1% dye concentration are significantly higher than the corresponding combed fabrics treated with 0.1% dye concentration (p<0.05). Similarly, this trend was found in combed Supima, twist, ESTex, 30Ne, 40Ne, reactive dyed, direct dyed, red, yellow and blue samples.

**Table 8:** Results of Post hoc tests showing the comparisons of fabrics treated with 0.1% dye, 1% dye and 5% dye under the interaction effect of other variables.

(I)	(J)	Mean Difference (I-J)	Std. Error	Sig.	95% Confidence Interval	
					Lower Bound	Upper Bound
0.1% dye-combed	1% dye-combed	-6.23426*	0.68387	0	-8.2587	-4.2098
0.1% dye-combed	5% dye-combed	-9.99284*	0.95562	0	-12.8324	-7.1533
1% dye-combed	5% dye-combed	-3.75857*	1.084	0.009	-6.9655	-0.5516
0.1% dye-combed Supima	1% dye-combed Supima	-2.74815*	0.64088	0	-4.6441	-0.8522
0.1% dye-combed Supima	5% dye-combed Supima	-5.87223*	0.85974	0	-8.4215	-3.323
1% dye-combed Supima	5% dye-combed Supima	-3.12408*	0.96799	0.021	-5.985	-0.2631
0.1% dye-Twist	1% dye-Twist	-5.32315*	0.75435	0	-7.5559	-3.0904
0.1% dye-Twist	5% dye-Twist	-8.24748*	0.98359	0	-11.1681	-5.3269
1% dye-Twist	5% dye-Twist	-2.92433	1.12977	0.143	-6.2649	0.4163
0.1% dye-ESTex	1% dye-ESTex	-3.65926*	0.57321	0	-5.3563	-1.9623
0.1% dye-ESTex	5% dye-ESTex	-7.47982*	0.86288	0	-10.0418	-4.9178
1% dye-ESTex	5% dye-ESTex	-3.82055*	0.96485	0.001	-6.6742	-0.9669
0.1% dye-30Ne yarn	1% dye-30Ne Yarn	-6.77410*	0.57003	0	-8.462	-5.0862
0.1% dye-30Ne yarn	5% dye-30Ne Yarn	-11.82182*	0.87743	0	-14.4298	-9.2138
1% dye-30Ne yarn	5% dye-30Ne Yarn	-5.04772*	0.98016	0	-7.9486	-2.1468
0.1% dye-40Ne yarn	1% dye-40Ne Yarn	-2.20832*	0.31222	0	-3.1338	-1.2828
0.1% dye-40Ne yarn	5% dye-40Ne Yarn	-4.05428*	0.38969	0	-5.2116	-2.897
1% dye-40Ne yarn	5% dye-40Ne Yarn	-1.84596*	0.46815	0.002	-3.2292	-0.4628
0.1%-reactive dye	1%-reactive dye	-5.13890*	0.81534	0	-7.7909	-2.4869
0.1%-reactive dye	5%-reactive dye	-11.58128*	1.29819	0	-15.8331	-7.3295
1%-reactive dye	5%-reactive dye	-6.44239*	1.43498	0.001	-11.1092	-1.7755
0.1%-direct dye	1%-direct dye	-6.43332*	0.8963	0	-9.3512	-3.5155
0.1%-direct dye	5%-direct dye	-9.02286*	1.07336	0	-12.5326	-5.5131
1%-direct dye	5%-direct dye	-2.58954	1.28186	0.809	-6.7463	1.5672
0.1%-sulphur dye	1%-sulphur dye	-1.90141	0.6012	0.064	-3.8491	0.0463
0.1%-sulphur dye	5%-sulphur dye	-3.23611*	0.65275	0	-5.3529	-1.1193
1%-sulphur dye	5%-sulphur dye	-1.33471	0.71113	0.901	-3.6374	0.968
0.1%-red	1%-red	-5.06807*	0.89945	0	-8.003	-2.1331
0.1%-red	5%-red	-7.58673*	1.13642	0	-11.3078	-3.8656
1%-red	5%-red	-2.51866	1.37126	0.921	-6.9645	1.9272
0.1%-yellow	1%-yellow	-4.41527*	0.84318	0	-7.1524	-1.6782
0.1%-yellow	5%-yellow	-6.08412*	1.04443	0	-9.4881	-2.6801
1%-yellow	5%-yellow	-1.66885	1.18087	0.998	-5.498	2.1603
0.1%-blue	1%-blue	-3.99028*	0.78906	0	-6.5534	-1.4271
0.1%-blue	5%-blue	-9.78787*	1.21017	0	-13.7464	-5.8294
1%-blue	5%-blue	-5.79759*	1.32739	0.001	-10.1133	-1.4818

* The mean difference is significant at the 0.05 level.

It is well known that fabrics with darker or more intense colour could provide better UV protection property. Contrary to previous finding, this study contends that the UPF value for some fabrics treated with 5% dye solution is lower than the one with 1% dye solution (e.g. Combed ESTex 30Ne Sulphur dye red, Combed twist 30Ne Direct dye yellow, Combed twist 30Ne Sulphur dye yellow, Combed ESTex 40Ne Direct dye yellow, Combed Supima twist 30Ne Sulphur dye yellow). This phenomenon is commonly found in the yellow samples and this can attribute to the reduction in fabric thickness on the 5% dyed fabrics. As shown in Table A in S1 File, the thickness of these 5% dyed samples is even lower than the corresponding 1% dyed fabrics. The effect of fabric thickness might override the effect of dye concentration in the yellow samples, resulting in unexpected finding observed. Overall, the UPF of the 5% dyed samples is the highest when compared with the 0.1% and 1% dyed samples. Dye on the fabric surface might absorb ultraviolet radiation in the visible and UV radiation band [22]. It reacts like additives to the fabric and improves UV protection abilities as they block UV transmission through the fabric to the skin. Dye concentration basically affects both the absorption and the reflectivity of UV photons by the textile material with dye molecules [12].

### Effect of dye class on UV protection

**Fig 9:**
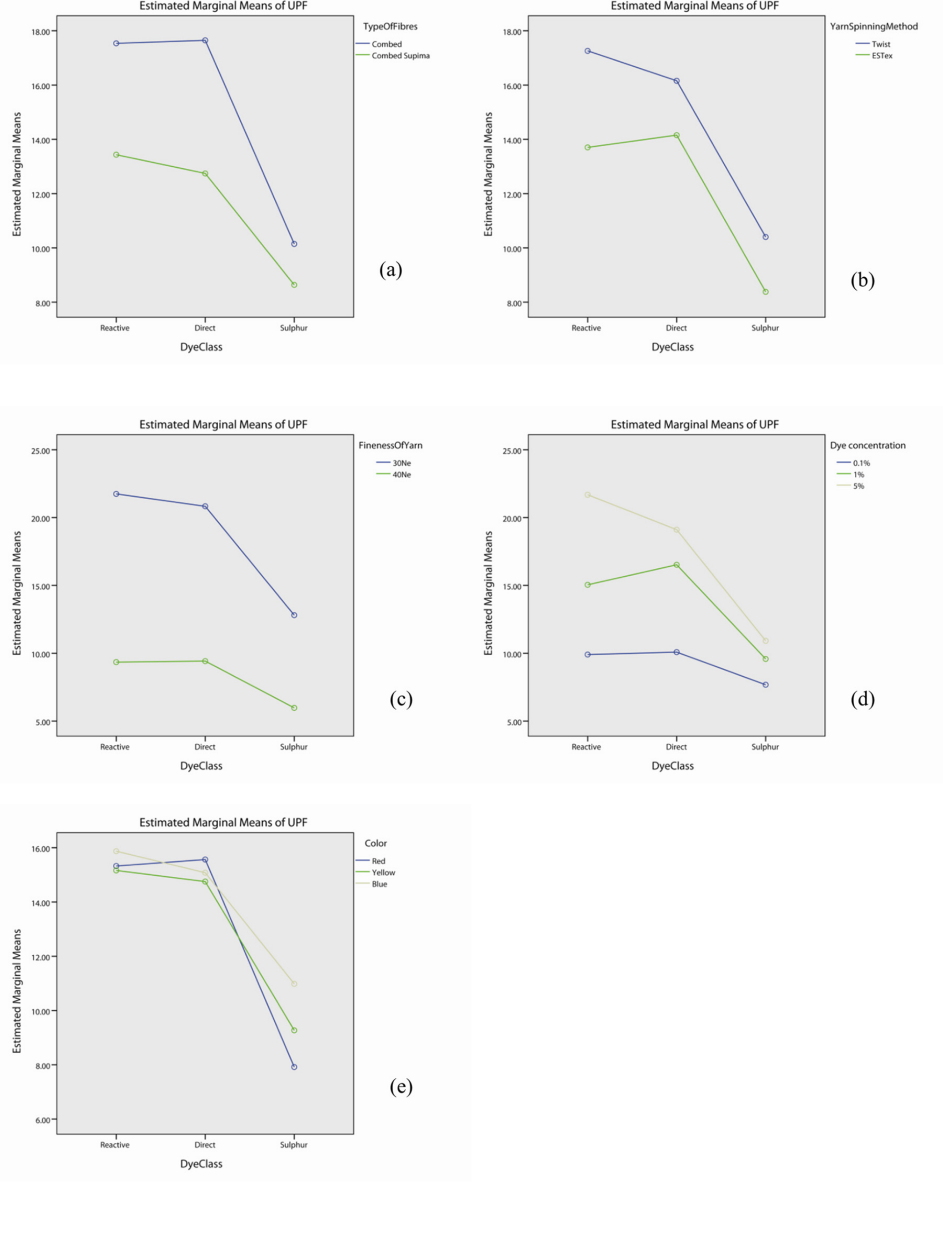
Profile plots showing the interaction effect of dye class with (a) types of fibre, (b) yarn spinning method, (c) yarn fineness, (d) dye concentration, and (e) colour.

As shown in Table F in S1 File, the interaction effect of dye class is significant with fibre type (p<0.05), yarn spinning method (p<0.05), yarn fineness (p<0.05), dye concentration (p<0.05) and colour (p<0.05). The profile plots of dye class with fibre type (Fig 9(A)), yarn spinning method (Fig 9(B)), fineness of yarn (Fig 9(C)), dye concentration (Fig 9(D)) and colour (Fig 9(E)) show that the UPF value of fabrics dyed with sulphur dye is generally lower than the one by reactive dye and sulphur dye. However, we could not simply say whether reactive dye or direct dye got high UPF value as this is actually type of fibre-dependent, yarn spinning method-dependent, yarn fineness-dependent, dye concentration-dependent and colour-dependent. Table F in S1 File also shows that the main effect of dye class is significant (p<0.05).

The post hoc tests, shown in Table 9, suggest that irrespective of fibre type, yarn spinning method, yarn fineness, dye concentration and colour, the UPF value of the fabrics dyed with sulphur dye is significantly lower than the corresponding fabrics by reactive dye and direct dye (p<0.05).

**Table 9:** Results of Post hoc tests showing the comparisons of fabrics made with reactive dye, direct dye and sulphur dye under the interaction effect of other variables.

(I)	(J)	Mean Difference (I-J)	Std. Error	Sig.	95% Confidence Interval	
					Lower Bound	Upper Bound
Reactive-combed	Direct dye-combed	-0.26505	1.08128	1	-3.4589	2.9288
Reactive-combed	Sulphur dye-combed	7.23761*	0.88633	0	4.6122	9.863
Direct dye-combed	Sulphur dye-combed	7.50266*	0.84323	0	5.0054	9.9999
Reactive-combed Supima	Direct dye-combed Supima	0.68795	0.96474	1	-2.1619	3.5378
Reactive-combed Supima	Sulphur dye-combed Supima	4.79721*	0.84757	0	2.2869	7.3076
Direct dye-combed Supima	Sulphur dye-combed Supima	4.10926*	0.70075	0	2.0371	6.1815
Reactive-Twist	Direct dye-Twist	1.10317	1.11141	0.997	-2.18	4.3863
Reactive-Twist	Sulphur dye-Twist	6.85813*	0.94552	0	4.0573	9.6589
Direct dye-Twist	Sulphur dye-Twist	5.75497*	0.83951	0	3.27	8.2399
Reactive-ESTex	Direct dye-ESTex	-0.6276	0.96918	1	-3.4893	2.2341
Reactive-ESTex	Sulphur dye-ESTex	5.14646*	0.78286	0	2.8273	7.4656
Direct dye-ESTex	Sulphur dye-ESTex	5.77406*	0.74592	0	3.5653	7.9828
Reactive-30Ne yarn	Direct dye-30Ne Yarn	0.78466	1.0152	1	-2.2149	3.7842
Reactive-30Ne yarn	Sulphur dye-30Ne Yarn	8.81147*	0.87064	0	6.2301	11.3928
Direct dye-30Ne yarn	Sulphur dye-30Ne Yarn	8.02681*	0.72433	0	5.8828	10.1709
Reactive-40Ne yarn	Direct dye-40Ne Yarn	-0.07464	0.46234	1	-1.4399	1.2906
Reactive-40Ne yarn	Sulphur dye-40Ne Yarn	3.37037*	0.35752	0	2.3109	4.4298
Direct dye-40Ne yarn	Sulphur dye-40Ne Yarn	3.44501*	0.36014	0	2.3774	4.5126
Reactive-0.1% dye	Direct-0.1% dye	-0.17918	0.54919	1	-1.9571	1.5987
Reactive-0.1% dye	Sulphur-0.1% dye	2.22777*	0.53513	0.002	0.4954	3.9602
Direct-0.1% dye	Sulphur-0.1% dye	2.40695*	0.54504	0.001	0.6424	4.1715
Reactive-1% dye	Direct-1% dye	-1.4736	1.08006	0.999	-4.9707	2.0235
Reactive-1% dye	Sulphur-1% dye	5.46526*	0.86015	0	2.6739	8.2566
Direct-1% dye	Sulphur-1% dye	6.93886*	0.93152	0	3.9123	9.9654
Reactive-5% dye	Direct-5% dye	2.37924	1.59242	0.995	-2.7848	7.5433
Reactive-5% dye	Sulphur-5% dye	10.57294*	1.35093	0	6.1634	14.9825
Direct-5% dye	Sulphur-5% dye	8.19370*	1.13187	0	4.5065	11.8809
Reactive dye-red	Direct dye-red	-0.23877	1.3417	1	-4.5837	4.1061
Reactive dye-red	Sulphur dye-red	7.40691*	0.99431	0	4.1542	10.6596
Direct dye-red	Sulphur dye-red	7.64568*	0.9868	0	4.4142	10.8772
Reactive dye-yellow	Direct dye-yellow	0.40808	1.18993	1	-3.4508	4.267
Reactive dye-yellow	Sulphur dye-yellow	5.89142*	1.03513	0	2.5129	9.2699
Direct dye-yellow	Sulphur dye-yellow	5.48334*	0.81894	0	2.8217	8.145
Reactive dye-blue	Direct dye-blue	0.54808	1.35347	1	-3.8359	4.932
Reactive dye-blue	Sulphur dye-blue	4.64301*	1.19304	0.005	0.7697	8.5164
Direct dye-blue	Sulphur dye-blue	4.09494*	1.10693	0.011	0.5034	7.6865

* The mean difference is significant at the 0.05 level.

UV transmittance and the corresponding UPF values for knitted cotton fabrics dyed with reactive dye, direct dye and sulphur dye are presented in Table 10. For the discussion of the effect of dye class on UV transmittance, fabrics with the same yarn count were selected (i.e. 30Ne). The undyed (control) fabric has on average 11.8% and 8.9% transmittance in UVA and UVB region, respectively. The UPF of these fabrics is 10.9 which are relatively low, so they may not be classified as UV protective fabrics. All three dye classes cause substantial reductions in UVA and UVB radiation transmittance which consequently results in the increase in the UPF values.

**Table 10:** UV Transmittance and the Corresponding UPF values for Cotton Knitted Fabrics Dyed with Reactive Dye, Direct Dye and Sulphur Dye with 0.1%, 1% and 5% owf (Using 30Ne Blue Colour fabrics as an explaining example).

Undyed (Control)			Transmittance (%) of fabrics								
			0.10%			1%			5%		
			UVA	UVB	UPF	UVA	UVB	UPF	UVA	UVB	UPF
			UVA = 11.8; UVB = 8.9; UPF = 10.9								
Reactive dye (R-B)	Combed	Twist	9.2	5.8	15.8	6.1	4.3	22.5	3.3	2.4	40.5
		ESTex	11.5	8	11.8	6.5	4.8	21.6	3.4	2.5	40.2
	Combed Supima	Twist	10.3	7.7	12.4	6.1	4.5	21.8	3.8	2.9	34.7
		ESTex	10.6	8.4	11.6	8.2	6.6	14.8	5.3	4.3	22.7
Direct dye (D-B)	Combed	Twist	9.1	7.5	12.5	4.2	3.8	25.3	2.9	2.9	37.9
		ESTex	8.4	6.9	13.6	4.7	4.2	23.4	3.3	3.1	32.8
	Combed Supima	Twist	8.8	7.5	13.5	5.8	5.3	18.9	4.9	4.5	21.7
		ESTex	11.7	10.2	9.5	6.4	5.8	17.1	5.6	5.3	19.5
Sulphur dye (S-B)	Combed	Twist	13.2	9.8	10.1	8.1	5.6	17.7	5.4	4.1	23.9
		ESTex	10	13.1	9.2	9	6.6	17.1	5.9	4.5	21.6
	Combed Supima	Twist	7.9	6	19.7	8.1	6.1	15.7	6.1	4.9	19.8
		ESTex	14.2	11.6	8.5	10.7	8.7	11.4	7.3	6	16.7

Direct and reactive dye can increase UPF of knitted samples which depends on relative transmittance of the dye in the UVB region. Generally speaking, all dyed fabrics showed considerable transmittance in the UVA region. However, because the relative erythemal spectral effectiveness is higher in the UVB region compared to the UVA region, UPF values depend primarily on transmission in the UVB region, based on the UPF equation [4]. Prior research [35] has also confirmed that some direct dyes are capable of providing a UPF of 50+ on textile materials. Good penetration and higher diffusion ability of direct dye contribute to the high UPF results. During the dyeing process, direct dye aggregates and then breaks down progressively into single molecules. Thus, the single molecules penetrate into the microspores of the cellulose fibres.

On the other hand, sulphur dye obtained relatively poor UPF results. The UPF results of sulphur dyed samples are even lower than control (undyed) samples. The average UPF of the sulphur dyed fabric is less than 25 and the highest UPF is only 23.87 at 5% owf. This result shows that sulphur dye cannot provide sufficient UV protection ability to textile fabrics. It is mainly due to the floating of liquid dye in the bath during the dyeing process, resulting in uneven colouring. Uneven colour appearance of dyed samples may contribute to the low UPF result of sulphur dyed samples. The effect of sulphur dyes on UVB transmittance is weak when compared with reactive and direct dye. The relative erythemal spectral effectiveness in UVB region is higher than in the UVA region. The UV transmittance of reactive dyed 5% owf fabrics in UVB region can be effectively reduced from 8.9% (control) to 2.4%, however, the UVB transmittance of the sulphur dyed 5% owf fabrics is still as low as 4.1%.

### Effect of colour on UV protection

**Fig 10:**
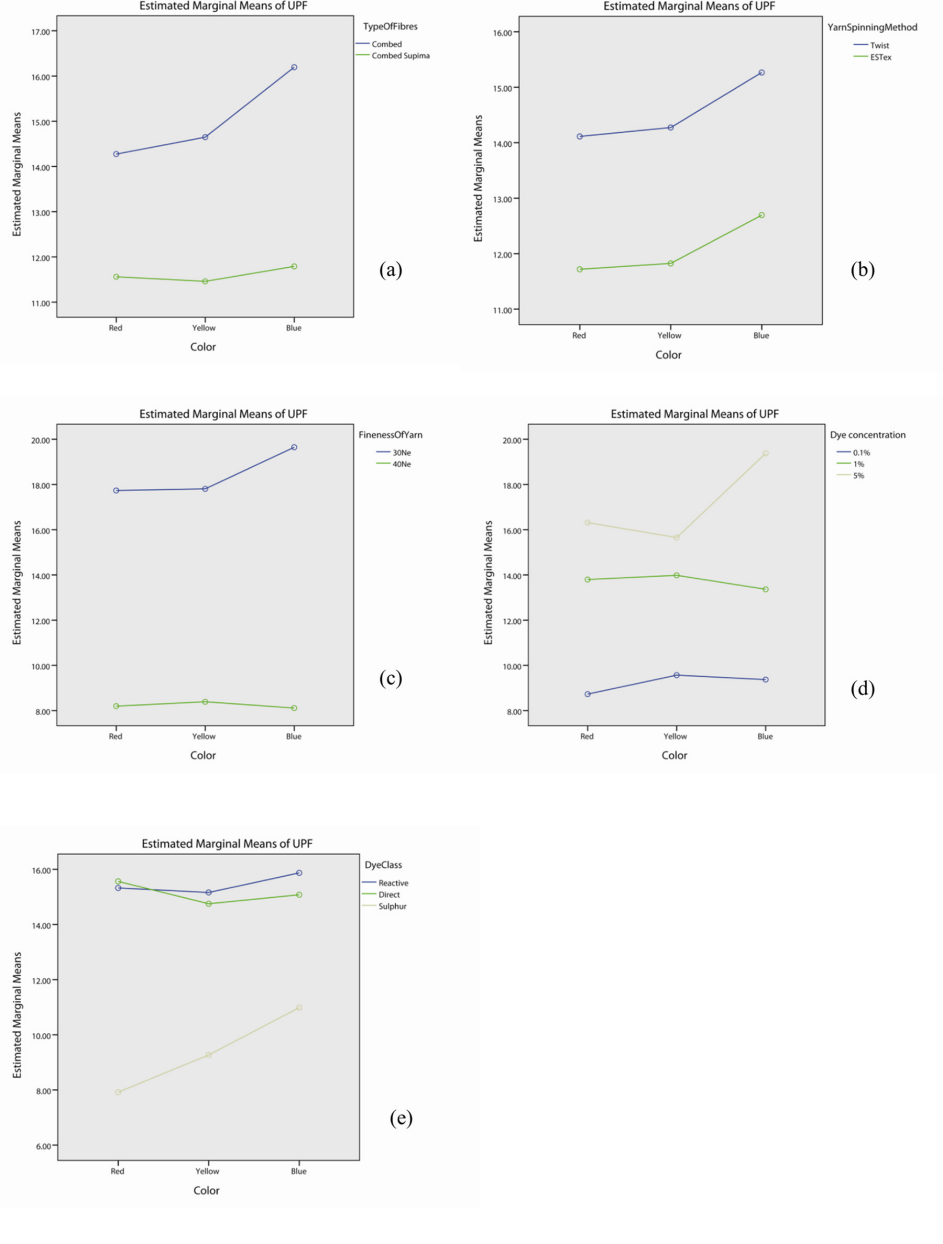
Profile plots showing the interaction effect of colour with (a) types of fibre, (b) yarn spinning method, (c) yarn fineness, (d) dye concentration, and (e) dye class.

The interaction effect of colour is significant with fibre type (p<0.05), yarn fineness (p<0.05), dye concentration (p<0.05) and dye class (p<0.05) but not with yarn spinning method (p>0.05). The profile plots of colour with fibre type (Fig 10(A)), fineness of yarn (Fig 10(C)), dye concentration (Fig 10(D)) and dye class (Fig 10(E)) show that the UPF value of fabrics is color-dependent. We cannot simply say which color is the best and which one is the worst. Table F in S1 File shows that the effect of colour is significant (p<0.05).

Post hoc tests, shown in Table 11, suggest that most of the interaction effect of colour and the other five variables is not significant (p>0.05). The only one exception is that the UPF of the blue-surplur dyed samples is significantly higher than the corresponding red-sulphur dyed samples (p<0.05).

**Table 11:** Results of Post hoc tests showing the comparisons of fabrics in red, yellow and blue colour under the interaction effect of other variables.

(I)	(J)	Mean Difference (I-J)	Std. Error	Sig.	95% Confidence Interval	
					Lower Bound	Upper Bound
Red-combed	Yellow-combed	-.37334	.95789	1.000	-3.2030	2.4563
Red-combed	Blue-combed	-1.75617	1.09087	.822	-4.9781	1.4657
Yellow-combed	Blue-combed	-1.38283	1.00969	.941	-4.3667	1.6010
Red-combed Supima	Yellow-combed Supima	.10095	.90249	1.000	-2.5638	2.7657
Red-combed Supima	Blue-combed Supima	-.22960	.88621	1.000	-2.8464	2.3872
Yellow-combed Supima	Blue-combed Supima	-.33056	.85304	1.000	-2.8492	2.1881
Red-Twist	Yellow-Twist	-.16093	.99957	1.000	-3.1136	2.7918
Red-Twist	Blue-Twist	-1.15331	1.08498	.994	-4.3575	2.0508
Yellow-Twist	Blue-Twist	-.99239	1.00245	.997	-3.9536	1.9688
Red-ESTex	Yellow-ESTex	-.10556	.87026	1.000	-2.6751	2.4640
Red-ESTex	Blue-ESTex	-.79127	.91437	.999	-3.4911	1.9085
Yellow-ESTex	Blue-ESTex	-.68571	.88811	1.000	-3.3082	1.9368
Red-30Ne yarn	Yellow-30Ne Yarn	-.07238	.99128	1.000	-3.0010	2.8562
Red-30Ne yarn	Blue-30Ne Yarn	-1.77880	1.04003	.750	-4.8503	1.2927
Yellow-30Ne yarn	Blue-30Ne Yarn	-1.70642	.93274	.655	-4.4610	1.0481
Red-40Ne yarn	Yellow-40Ne Yarn	-.19165	.44112	1.000	-1.4940	1.1107
Red-40Ne yarn	Blue-40Ne Yarn	.08480	.43805	1.000	-1.2087	1.3783
Yellow-40Ne yarn	Blue-40Ne Yarn	.27645	.43647	1.000	-1.0124	1.5653
Red-0.1% dye	Yellow-0.1% dye	-.84028	.56011	.995	-2.6559	.9754
Red-0.1% dye	Blue-0.1% dye	-.64444	.52214	1.000	-2.3357	1.0468
Yellow-0.1% dye	Blue-0.1% dye	.19584	.60518	1.000	-1.7637	2.1554
Red-1% dye	Yellow-1% dye	-.18748	1.09829	1.000	-3.7443	3.3694
Red-1% dye	Blue-1% dye	.43335	1.07657	1.000	-3.0540	3.9208
Yellow-1% dye	Blue-1% dye	.62083	.98353	1.000	-2.5633	3.8049
Red-5% dye	Yellow-5% dye	.66233	1.43825	1.000	-3.9983	5.3229
Red-5% dye	Blue-5% dye	-2.84558	1.57586	.934	-7.9512	2.2600
Yellow-5% dye	Blue-5% dye	-3.50790	1.47956	.496	-8.3041	1.2883
Red-reactive dye	Yellow-reactive dye	.16413	1.34755	1.000	-4.1998	4.5280
Red-reactive dye	Blue-reactive dye	-.30134	1.38653	1.000	-4.7906	4.1879
Yellow-reactive dye	Blue-reactive dye	-.46547	1.38685	1.000	-4.9570	4.0261
Red-direct dye	Yellow-direct dye	.81098	1.18330	1.000	-3.0261	4.6481
Red-direct dye	Blue-direct dye	.48551	1.30750	1.000	-3.7502	4.7212
Yellow-direct dye	Blue-direct dye	-.32547	1.15086	1.000	-4.0560	3.4051
Red-sulphur dye	Yellow-sulphur dye	-1.35135	.49420	.221	-2.9539	.2512
Red-sulphur dye	Blue-sulphur dye	-3.06523*	.69966	.001	-5.3454	-.7851
Yellow-sulphur dye	Blue-sulphur dye	-1.71388	.75597	.594	-4.1679	.7402

* The mean difference is significant at the 0.05 level.

### Relationship between CIE L* of fabric and UV protection

Wilson et al. (2008) [12] investigated the relationship between fabric colour and UV transmittance and concluded that the effect of dye concentration is more important in affecting UV transmittance than colour shade. The L* components of the CIE L*a*b*system can be the best description of dye concentration. It represents the lightness of the colour (CIE L* = 0 yields black colour and CIE L* = 100 indicates diffuse white).

**Fig 11:**
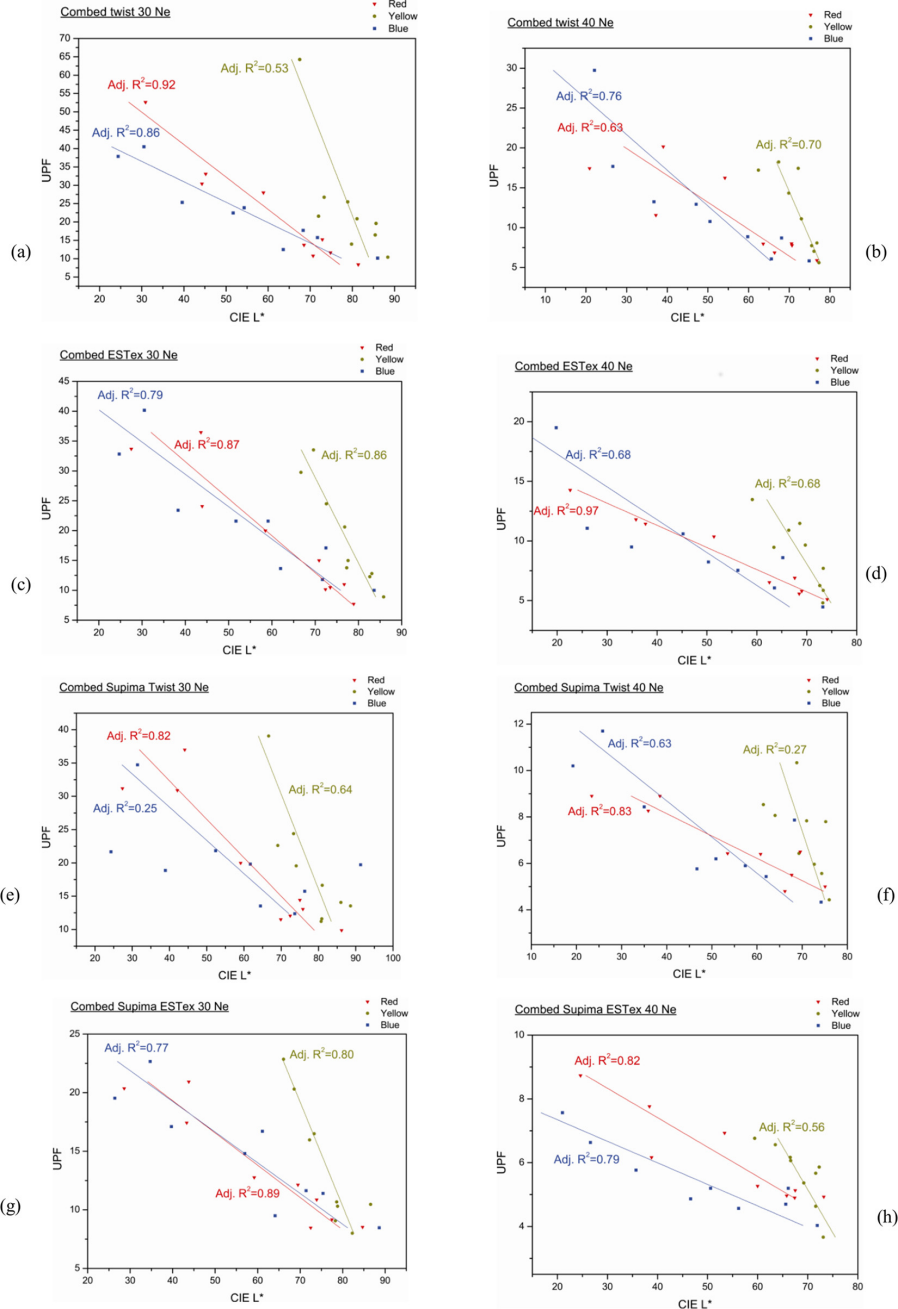
Correlation between CIE L* and UPF for (a) Combed twist 30Ne fabrics, (b) Combed twist 40Ne fabrics, (c) Combed ESTex 30Ne fabrics, (d) Combed ESTex 40Ne fabrics, (e) Combed Supima twist 30Ne fabrics, (f) Combed Supima twist 40Ne fabrics, (g) Combed Supima ESTex 30Ne fabrics, (h) Combed Supima ESTex 40Ne fabrics.

The relationship between lightness of fabrics and UPF is shown in Fig 11. It can be observed that UPF value is negatively related to L* value and is dependent on dye concentration. The lighter the colour of the fabric, the lower the UPF value is. For example, in case of combed twist 30Ne fabric samples dyed with reactive red dye, its CIE L* value decreases from 74.8 to 58.9 and then further to 44.3 when dye concentration increases from 0.1% to 1% and then to 5%. Fig 2 shows that UPF value of this fabric (i.e. combed twist 30Ne reactive dyed red fabric) increases from 11.67 to 30.40 when dye concentration increases from 0.1% to 5%. CIE L* value affects UV transmittance in both UVA and UVB regions as dye concentration affects both the reflectivity and the absorption of UV photons when the dye molecules are present on the fabric. When developing and choosing fabrics with UV protection ability, dyes that generate colours with small CIE L* values are recommended. However, colour must be considered in combination with other physical properties known to enhance the UV transmission.

## Conclusions

In this study, plain cotton knitted fabrics made from different fibre types (combed cotton and combed Supima cotton), yarn types (conventional ring spun yarn and torque-free ring spun yarn), yarn fineness (30Ne and 40Ne) and dyed with three dye classes (reactive, direct and sulphur dye) with three dye concentrations (0.1%, 1.0% and 5.0%) in three different colours (red, yellow, blue) were tested for UV properties. ANOVA test suggests that both the main effect and interaction effect of these variables is significant in affecting UPF property. The F-ratio of ANOVA test shows that the magnitude of the main effect is great and is higher than the 2-way interaction effect. Fineness of yarn is the most significant factor varying the UPF (F = 3062.2), followed by dye concentration (F = 656.6), dye class (F = 486.6), types of fibers (F = 409.5), yarn spinning method (F = 234.2) and color (F = 6.9). Experimental results revealed that the UPF value of dyed fabrics made from combed Supima cotton is generally lower than the combed cotton since combed cotton is composed of shorter fibres which facilitate the blocking or absorption of UV radiation. Apart from that, fabrics made with twist yarn (i.e. ring spun yarn) have higher UPF value than the corresponding ESTex one (i.e. torque-free yarn) in most of the cases. The conventional ring-spun yarn has more twist than the torque-free ring spun yarn, during wet treatment with dyeing, the residual torque in the yarn is released from the conventional ring spun yarn and the fabric becomes distorted and shrinks. Thus, the structure of knitted fabrics made from conventional ring spun yarn becomes more compact which helps resist penetration of UV rays. When the yarn properties were taken into consideration, UPF value of fabrics made of 30Ne yarn was higher than the 40Ne one irrespective of dye class and colour. This is because fabrics made with 30Ne yarn are thicker and have lower fabric porosity than the 40Ne one.

For the effect of dye, fabrics with 0.1% dye concentration gave the lowest UPF in all dye classes while the sulphur dyed samples performed worse than the reactive and direct dyed samples no matter what yarn or fibre was used. Partial Eta squared for the effect of colour is the lowest and only 1.7% of the variance in UPF can be predicted from colour. There is no significant difference in UPF for red, yellow and blue coloured fabrics. This study also demonstrated that lightness of fabric is negatively related to UV protection property.

## Supporting Information

S1 File Supporting information. (DOC)
