# Supplementary material for: In-Vitro Analysis of the Effect of Constructional Parameters and Dye Class on the UV Protection Property of Cotton Knitted Fabrics
Source: PLoS One. 2015 Jul 29;10(7):e0133416. doi: 10.1371/journal.pone.0133416 (PMC4519329; doi:10.1371/journal.pone.0133416)
Supplement: S1 File — (DOC) [file pone.0133416.s001.doc]

Table A. Thickness (mm) of dyed fabrics

|  | | | | Reactive dye | | | Direct dye | | | Sulphur dye | | |
| --- | --- | --- | --- | --- | --- | --- | --- | --- | --- | --- | --- | --- |
|  |  |  |  | Red | Yellow | Blue | Red | Yellow | Blue | Red | Yellow | Blue |
|  |  |  |  | R-R | R-Y | R-B | D-R | D-Y | D-B | S-R | S-Y | S-B |
| Combed | Twist | 30Ne | 0.1 % | 1.009 | 1.093 | 1.036 | 1.029 | 1.111 | 1.032 | 1.084 | 1.110 | 1.005 |
|  |  |  | 1 % | 1.085 | 1.068 | 1.058 | 1.038 | 1.088 | 1.061 | 1.080 | 1.121 | 1.046 |
|  |  |  | 5 % | 1.025 | 1.079 | 1.053 | 1.075 | 1.046 | 1.042 | 1.064 | 1.060 | 1.030 |
|  |  | 40Ne | 0.1 % | 0.919 | 0.955 | 0.970 | 0.938 | 0.978 | 0.976 | 0.980 | 0.951 | 1.051 |
|  |  |  | 1 % | 0.924 | 0.971 | 0.954 | 0.975 | 0.969 | 0.951 | 0.954 | 0.948 | 0.899 |
|  |  |  | 5 % | 0.894 | 0.976 | 0.926 | 0.960 | 0.938 | 1.073 | 0.955 | 0.949 | 0.969 |
|  | ESTex | 30Ne | 0.1 % | 0.995 | 0.981 | 1.000 | 0.986 | 0.959 | 0.901 | 0.990 | 1.021 | 0.964 |
|  |  |  | 1 % | 0.958 | 1.100 | 0.944 | 0.963 | 0.950 | 0.923 | 1.010 | 0.975 | 0.953 |
|  |  |  | 5 % | 0.980 | 0.989 | 0.976 | 0.999 | 0.971 | 0.931 | 0.984 | 0.970 | 0.961 |
|  |  | 40Ne | 0.1 % | 0.863 | 0.859 | 0.863 | 0.890 | 0.928 | 0.870 | 0.898 | 0.893 | 0.886 |
|  |  |  | 1 % | 0.876 | 0.888 | 0.879 | 0.909 | 0.924 | 0.870 | 0.888 | 0.864 | 0.858 |
|  |  |  | 5 % | 0.870 | 0.913 | 0.808 | 0.899 | 0.895 | 0.921 | 0.895 | 0.900 | 0.928 |
| Combed Supima | Twist | 30Ne | 0.1 % | 1.014 | 1.009 | 0.990 | 0.948 | 1.038 | 0.968 | 0.988 | 1.045 | 0.984 |
|  |  |  | 1 % | 0.999 | 1.055 | 1.015 | 1.014 | 1.020 | 0.955 | 1.009 | 1.046 | 1.008 |
|  |  |  | 5 % | 1.023 | 1.031 | 0.989 | 1.012 | 0.949 | 0.901 | 1.003 | 0.981 | 1.004 |
|  |  | 40Ne | 0.1 % | 0.863 | 0.875 | 0.869 | 0.915 | 0.900 | 0.901 | 0.900 | 0.875 | 0.885 |
|  |  |  | 1 % | 0.879 | 0.893 | 0.863 | 0.899 | 0.884 | 0.903 | 0.918 | 0.873 | 0.841 |
|  |  |  | 5 % | 0.863 | 0.931 | 0.839 | 0.883 | 0.873 | 0.883 | 0.913 | 0.894 | 0.925 |
|  | ESTex | 30Ne | 0.1 % | 0.868 | 0.933 | 0.891 | 0.886 | 0.848 | 0.815 | 0.906 | 0.910 | 0.848 |
|  |  |  | 1 % | 0.905 | 0.928 | 0.868 | 0.916 | 0.872 | 0.831 | 0.901 | 0.876 | 0.860 |
|  |  |  | 5 % | 0.871 | 0.911 | 0.895 | 0.881 | 0.848 | 0.849 | 0.898 | 0.854 | 0.865 |
|  |  | 40Ne | 0.1 % | 0.764 | 0.783 | 0.730 | 0.770 | 0.834 | 0.776 | 0.799 | 0.774 | 0.784 |
|  |  |  | 1 % | 0.808 | 0.819 | 0.754 | 0.778 | 0.808 | 0.783 | 0.811 | 0.761 | 0.760 |
|  |  |  | 5 % | 0.775 | 0.808 | 0.714 | 0.783 | 0.828 | 0.778 | 0.785 | 0.765 | 0.808 |

Table B. Porosity of dyed fabrics

|  | | | | Reactive dye | | | Direct dye | | | Sulphur dye | | |
| --- | --- | --- | --- | --- | --- | --- | --- | --- | --- | --- | --- | --- |
|  |  |  |  | Red | Yellow | Blue | Red | Yellow | Blue | Red | Yellow | Blue |
|  |  |  |  | R-R | R-Y | R-B | D-R | D-Y | D-B | S-R | S-Y | S-B |
| Combed | Twist | 30Ne | 0.1 % | 0.883 | 0.892 | 0.883 | 0.882 | 0.890 | 0.887 | 0.894 | 0.899 | 0.881 |
|  |  |  | 1 % | 0.897 | 0.897 | 0.885 | 0.890 | 0.891 | 0.890 | 0.892 | 0.893 | 0.889 |
|  |  |  | 5 % | 0.897 | 0.872 | 0.889 | 0.883 | 0.891 | 0.887 | 0.893 | 0.898 | 0.882 |
|  |  | 40Ne | 0.1 % | 0.905 | 0.908 | 0.910 | 0.909 | 0.911 | 0.905 | 0.913 | 0.907 | 0.918 |
|  |  |  | 1 % | 0.899 | 0.907 | 0.910 | 0.911 | 0.901 | 0.906 | 0.914 | 0.910 | 0.910 |
|  |  |  | 5 % | 0.901 | 0.908 | 0.906 | 0.908 | 0.911 | 0.911 | 0.909 | 0.907 | 0.914 |
|  | ESTex | 30Ne | 0.1 % | 0.901 | 0.878 | 0.897 | 0.885 | 0.883 | 0.879 | 0.891 | 0.896 | 0.886 |
|  |  |  | 1 % | 0.891 | 0.914 | 0.876 | 0.890 | 0.888 | 0.881 | 0.898 | 0.891 | 0.888 |
|  |  |  | 5 % | 0.890 | 0.895 | 0.880 | 0.887 | 0.885 | 0.881 | 0.901 | 0.893 | 0.888 |
|  |  | 40Ne | 0.1 % | 0.907 | 0.908 | 0.907 | 0.913 | 0.911 | 0.913 | 0.915 | 0.913 | 0.913 |
|  |  |  | 1 % | 0.910 | 0.907 | 0.911 | 0.913 | 0.909 | 0.911 | 0.914 | 0.910 | 0.910 |
|  |  |  | 5 % | 0.908 | 0.910 | 0.909 | 0.907 | 0.916 | 0.910 | 0.915 | 0.908 | 0.915 |
| Combed Supima | Twist | 30Ne | 0.1 % | 0.883 | 0.874 | 0.880 | 0.879 | 0.882 | 0.877 | 0.881 | 0.886 | 0.888 |
|  |  |  | 1 % | 0.881 | 0.900 | 0.882 | 0.880 | 0.882 | 0.877 | 0.883 | 0.885 | 0.888 |
|  |  |  | 5 % | 0.884 | 0.893 | 0.873 | 0.881 | 0.882 | 0.874 | 0.879 | 0.889 | 0.878 |
|  |  | 40Ne | 0.1 % | 0.907 | 0.900 | 0.897 | 0.908 | 0.908 | 0.911 | 0.911 | 0.910 | 0.915 |
|  |  |  | 1 % | 0.909 | 0.905 | 0.908 | 0.910 | 0.896 | 0.909 | 0.913 | 0.901 | 0.908 |
|  |  |  | 5 % | 0.903 | 0.907 | 0.902 | 0.908 | 0.904 | 0.904 | 0.913 | 0.904 | 0.911 |
|  | ESTex | 30Ne | 0.1 % | 0.893 | 0.876 | 0.875 | 0.887 | 0.877 | 0.880 | 0.885 | 0.884 | 0.880 |
|  |  |  | 1 % | 0.882 | 0.880 | 0.879 | 0.890 | 0.881 | 0.878 | 0.885 | 0.891 | 0.875 |
|  |  |  | 5 % | 0.881 | 0.891 | 0.880 | 0.881 | 0.878 | 0.881 | 0.890 | 0.888 | 0.883 |
|  |  | 40Ne | 0.1 % | 0.903 | 0.908 | 0.902 | 0.904 | 0.908 | 0.911 | 0.906 | 0.912 | 0.909 |
|  |  |  | 1 % | 0.907 | 0.909 | 0.912 | 0.909 | 0.913 | 0.910 | 0.910 | 0.908 | 0.905 |
|  |  |  | 5 % | 0.908 | 0.907 | 0.903 | 0.903 | 0.905 | 0.901 | 0.908 | 0.908 | 0.907 |

Table C. Descriptive data summarising the distribution of UPF value of each group

|  |  | Skewness | | Kurtosis | |
| --- | --- | --- | --- | --- | --- |
|  |  | Statistics | Std. Error | Statistics | Std. Error |
| Types of fibres | Combed | 1.174 | 0.119 | 0.802 | 0.238 |
|  | Combed supima | 1.568 | 0.117 | 2.457 | 0.234 |
| Yarn spinning method | Twist | 1.171 | 0.119 | 0.849 | 0.238 |
|  | ESTex | 1.532 | 0.118 | 2.256 | 0.235 |
| Fineness of yarn | 30Ne | 0.949 | 0.119 | 0.150 | 0.237 |
|  | 40Ne | 1.435 | 0.118 | 1.584 | 0.235 |
| Dye concentration | 0.1 % | 0.827 | 0.144 | 0.348 | 0.286 |
|  | 1 % | 0.654 | 0.144 | -0.443 | 0.286 |
|  | 5 % | 0.856 | 0.147 | -0.355 | 0.293 |
| Dye class | Reactive dye | 1.159 | 0.145 | 0.549 | 0.289 |
|  | Direct dye | 0.906 | 0.146 | 0.119 | 0.290 |
|  | Sulphur dye | 1.107 | 0.144 | 0.699 | 0.286 |
| Color | Red | 1.460 | 0.145 | 1.295 | 0.288 |
|  | Yellow | 1.204 | 0.145 | 1.302 | 0.288 |
|  | Blue | 1.291 | 0.145 | 1.505 | 0.289 |

**Table D. Levene's Test of Equality of Error Variances**

| Dependent Variable: UPF | | | |
| --- | --- | --- | --- |
| F | df1 | df2 | Sig. |
| 2.823 | 212 | 638 | .000 |

Tests the null hypothesis that the error variance of the dependent variable is equal across groups.

Table E. ANOVA test results - Tests of Between-Subjects Effects

| Dependent Variable:UPF | | | | | | |
| --- | --- | --- | --- | --- | --- | --- |
| Source | Type III Sum of Squares | df | Mean Square | F | Sig. | Partial Eta Squared |
| Corrected Model | 56391.487^a^ | 212 | 265.998 | 344.407 | .000 | .991 |
| Intercept | 151147.671 | 1 | 151147.671 | 195702.3 | .000 | .997 |
| TypeOfFibre | 3124.803 | 1 | 3124.803 | 4045.919 | .000 | .864 |
| YarnSpinningMethod | 1769.130 | 1 | 1769.130 | 2290.626 | .000 | .782 |
| FinenessOfYarn | 20742.435 | 1 | 20742.435 | 26856.80 | .000 | .977 |
| DyeConcentration | 9640.648 | 2 | 4820.324 | 6241.238 | .000 | .951 |
| DyeClass | 7198.956 | 2 | 3599.478 | 4660.516 | .000 | .936 |
| Color | 150.573 | 2 | 75.286 | 97.479 | .000 | .234 |
| TypeOfFibre * YarnSpinningMethod | 53.729 | 1 | 53.729 | 69.568 | .000 | .098 |
| TypeOfFibre * FinenessOfYarn | 3.327 | 1 | 3.327 | 4.308 | .038 | .007 |
| TypeOfFibre * DyeConcentration | 993.046 | 2 | 496.523 | 642.886 | .000 | .668 |
| TypeOfFibre * DyeClass | 618.837 | 2 | 309.419 | 400.628 | .000 | .557 |
| TypeOfFibre * Color | 69.636 | 2 | 34.818 | 45.081 | .000 | .124 |
| YarnSpinningMethod * FinenessOfYarn | 102.689 | 1 | 102.689 | 132.959 | .000 | .172 |
| YarnSpinningMethod * DyeConcentration | 141.401 | 2 | 70.700 | 91.541 | .000 | .223 |
| YarnSpinningMethod * DyeClass | 125.351 | 2 | 62.675 | 81.150 | .000 | .203 |
| YarnSpinningMethod * Color | .784 | 2 | .392 | .508 | .602 | .002 |
| FinenessOfYarn * DyeConcentration | 1984.915 | 2 | 992.458 | 1285.010 | .000 | .801 |
| FinenessOfYarn * DyeClass | 1276.321 | 2 | 638.161 | 826.275 | .000 | .721 |
| FinenessOfYarn * Color | 42.577 | 2 | 21.288 | 27.564 | .000 | .080 |
| DyeConcentration * DyeClass | 2176.672 | 4 | 544.168 | 704.576 | .000 | .815 |
| DyeConcentration * Color | 434.878 | 4 | 108.720 | 140.767 | .000 | .469 |
| DyeClass * Color | 396.426 | 4 | 99.107 | 128.321 | .000 | .446 |
| TypeOfFibre * YarnSpinningMethod * FinenessOfYarn | 337.742 | 1 | 337.742 | 437.300 | .000 | .407 |
| TypeOfFibre * YarnSpinningMethod * DyeConcentration | 14.110 | 2 | 7.055 | 9.135 | .000 | .028 |
| TypeOfFibre * YarnSpinningMethod * DyeClass | 5.277 | 2 | 2.639 | 3.417 | .033 | .011 |
| TypeOfFibre * YarnSpinningMethod * Color | .152 | 2 | .076 | .098 | .906 | .000 |
| TypeOfFibre * FinenessOfYarn * DyeConcentration | 10.342 | 2 | 5.171 | 6.695 | .001 | .021 |
| TypeOfFibre * FinenessOfYarn * DyeClass | 1.661 | 2 | .831 | 1.075 | .342 | .003 |
| TypeOfFibre * FinenessOfYarn * Color | 3.989 | 2 | 1.994 | 2.582 | .076 | .008 |
| TypeOfFibre * DyeConcentration * DyeClass | 66.242 | 4 | 16.560 | 21.442 | .000 | .119 |
| TypeOfFibre * DyeConcentration * Color | 162.788 | 4 | 40.697 | 52.693 | .000 | .248 |
| TypeOfFibre * DyeClass * Color | 67.467 | 4 | 16.867 | 21.839 | .000 | .120 |
| YarnSpinningMethod * FinenessOfYarn * DyeConcentration | 63.422 | 2 | 31.711 | 41.058 | .000 | .114 |
| YarnSpinningMethod * FinenessOfYarn * DyeClass | 60.990 | 2 | 30.495 | 39.484 | .000 | .110 |
| YarnSpinningMethod * FinenessOfYarn * Color | 26.734 | 2 | 13.367 | 17.307 | .000 | .051 |
| YarnSpinningMethod * DyeConcentration * DyeClass | 69.793 | 4 | 17.448 | 22.591 | .000 | .124 |
| YarnSpinningMethod * DyeConcentration * Color | 124.997 | 4 | 31.249 | 40.461 | .000 | .202 |
| YarnSpinningMethod * DyeClass * Color | 27.973 | 4 | 6.993 | 9.055 | .000 | .054 |
| FinenessOfYarn * DyeConcentration * DyeClass | 1085.739 | 4 | 271.435 | 351.447 | .000 | .688 |
| FinenessOfYarn * DyeConcentration * Color | 90.781 | 4 | 22.695 | 29.385 | .000 | .156 |
| FinenessOfYarn * DyeClass * Color | 521.178 | 4 | 130.295 | 168.702 | .000 | .514 |
| DyeConcentration * DyeClass * Color | 193.330 | 8 | 24.166 | 31.290 | .000 | .282 |
| TypeOfFibre * YarnSpinningMethod * FinenessOfYarn * DyeConcentration | 156.478 | 2 | 78.239 | 101.302 | .000 | .241 |
| TypeOfFibre * YarnSpinningMethod * FinenessOfYarn * DyeClass | 39.952 | 2 | 19.976 | 25.865 | .000 | .075 |
| TypeOfFibre * YarnSpinningMethod * FinenessOfYarn * Color | 8.270 | 2 | 4.135 | 5.354 | .005 | .017 |
| TypeOfFibre * YarnSpinningMethod * DyeConcentration * DyeClass | 126.633 | 4 | 31.658 | 40.990 | .000 | .204 |
| TypeOfFibre * YarnSpinningMethod * DyeConcentration * Color | 72.561 | 4 | 18.140 | 23.487 | .000 | .128 |
| TypeOfFibre * YarnSpinningMethod * DyeClass * Color | 37.319 | 4 | 9.330 | 12.080 | .000 | .070 |
| TypeOfFibre * FinenessOfYarn * DyeConcentration * DyeClass | 88.186 | 4 | 22.046 | 28.545 | .000 | .152 |
| TypeOfFibre * FinenessOfYarn * DyeConcentration * Color | 46.754 | 4 | 11.688 | 15.134 | .000 | .087 |
| TypeOfFibre * FinenessOfYarn * DyeClass * Color | 18.666 | 4 | 4.667 | 6.042 | .000 | .036 |
| TypeOfFibre * DyeConcentration * DyeClass * Color | 171.264 | 8 | 21.408 | 27.719 | .000 | .258 |
| YarnSpinningMethod * FinenessOfYarn * DyeConcentration * DyeClass | 23.521 | 4 | 5.880 | 7.614 | .000 | .046 |
| YarnSpinningMethod * FinenessOfYarn * DyeConcentration * Color | 85.657 | 4 | 21.414 | 27.727 | .000 | .148 |
| YarnSpinningMethod * FinenessOfYarn * DyeClass * Color | 107.481 | 4 | 26.870 | 34.791 | .000 | .179 |
| YarnSpinningMethod * DyeConcentration * DyeClass * Color | 65.398 | 8 | 8.175 | 10.584 | .000 | .117 |
| FinenessOfYarn * DyeConcentration * DyeClass * Color | 172.144 | 8 | 21.518 | 27.861 | .000 | .259 |
| TypeOfFibre * YarnSpinningMethod * FinenessOfYarn * DyeConcentration * DyeClass | 270.860 | 4 | 67.715 | 87.676 | .000 | .355 |
| TypeOfFibre * YarnSpinningMethod * FinenessOfYarn * DyeConcentration * Color | 33.626 | 4 | 8.407 | 10.885 | .000 | .064 |
| TypeOfFibre * YarnSpinningMethod * FinenessOfYarn * DyeClass * Color | 68.411 | 4 | 17.103 | 22.144 | .000 | .122 |
| TypeOfFibre * YarnSpinningMethod * DyeConcentration * DyeClass * Color | 81.012 | 8 | 10.126 | 13.111 | .000 | .141 |
| TypeOfFibre * FinenessOfYarn * DyeConcentration * DyeClass * Color | 187.310 | 8 | 23.414 | 30.316 | .000 | .275 |
| YarnSpinningMethod * FinenessOfYarn * DyeConcentration * DyeClass * Color | 165.909 | 8 | 20.739 | 26.852 | .000 | .252 |
| TypeOfFibre *YarnSpinningMethod*FinenessOfYarn*DyeConcentration*DyeClass*Color | 55.314 | 5 | 11.063 | 14.324 | .000 | .101 |
| Error | 492.749 | 638 | .772 |  |  |  |
| Total | 206672.156 | 851 |  |  |  |  |
| Corrected Total | 56884.236 | 850 |  |  |  |  |
| a. R Squared = .991 (Adjusted R Squared = .988)  b. Computed using alpha = .05 | | | | | | |

Table F. Pooled ANOVA test results - Tests of Between-Subjects Effects

| Dependent Variable:UPF | | | | | | |
| --- | --- | --- | --- | --- | --- | --- |
| Source | Type III Sum of Squares | df | Mean Square | F | Sig. | Partial Eta Squared |
| Corrected Model | 50868.457a | 42 | 1211.154 | 162.674 | .000 | .894 |
| Intercept | 154501.999 | 1 | 154501.999 | 20751.696 | .000 | .963 |
| TypeOfFibres | 3048.829 | 1 | 3048.829 | 409.499 | .000 | .336 |
| YarnSpinningMethod | 1743.928 | 1 | 1743.928 | 234.233 | .000 | .225 |
| FinenessOfYarn | 22799.002 | 1 | 22799.002 | 3062.213 | .000 | .791 |
| DyeConcentration | 9776.515 | 2 | 4888.258 | 656.559 | .000 | .619 |
| DyeClass | 7245.640 | 2 | 3622.820 | 486.593 | .000 | .546 |
| Color | 102.926 | 2 | 51.463 | 6.912 | .001 | .017 |
| DyeConcentration * Color | 345.126 | 4 | 86.281 | 11.589 | .000 | .054 |
| DyeClass * Color | 481.904 | 4 | 120.476 | 16.182 | .000 | .074 |
| FinenessOfYarn * Color | 82.373 | 2 | 41.186 | 5.532 | .004 | .014 |
| TypeOfFibres * Color | 47.497 | 2 | 23.748 | 3.190 | .042 | .008 |
| YarnSpinningMethod * Color | 5.381 | 2 | 2.690 | .361 | .697 | .001 |
| DyeConcentration * DyeClass | 2260.211 | 4 | 565.053 | 75.894 | .000 | .273 |
| FinenessOfYarn * DyeConcentration | 2373.435 | 2 | 1186.717 | 159.392 | .000 | .283 |
| TypeOfFibres * DyeConcentration | 909.906 | 2 | 454.953 | 61.106 | .000 | .131 |
| YarnSpinningMethod * DyeConcentration | 118.946 | 2 | 59.473 | 7.988 | .000 | .019 |
| FinenessOfYarn * DyeClass | 1392.176 | 2 | 696.088 | 93.494 | .000 | .188 |
| TypeOfFibres * DyeClass | 575.647 | 2 | 287.823 | 38.659 | .000 | .087 |
| YarnSpinningMethod * DyeClass | 168.773 | 2 | 84.386 | 11.334 | .000 | .027 |
| TypeOfFibres * FinenessOfYarn | .325 | 1 | .325 | .044 | .835 | .000 |
| YarnSpinningMethod * FinenessOfYarn | 187.945 | 1 | 187.945 | 25.243 | .000 | .030 |
| TypeOfFibres * YarnSpinningMethod | 99.150 | 1 | 99.150 | 13.317 | .000 | .016 |
| Error | 6015.779 | 808 | 7.445 |  |  |  |
| Total | 206672.156 | 851 |  |  |  |  |
| Corrected Total | 56884.236 | 850 |  |  |  |  |
| a. R Squared = .894 (Adjusted R Squared = .889) | | | | | | |
